# ﻿Monstrilloid copepods (Crustacea, Copepoda) in the U.S. National Museum of Natural History, Smithsonian Institution: updated redescriptions of Mexican species of *Monstrilla* Dana

**DOI:** 10.3897/zookeys.1251.157981

**Published:** 2025-09-10

**Authors:** Eduardo Suárez-Morales

**Affiliations:** 1 El Colegio de la Frontera Sur (ECOSUR), Unidad Chetumal. Av. Centenario Km. 5.5, Chetumal, Quintana Roo 77014, Mexico El Colegio de la Frontera Sur Chetumal Mexico; † Deceased El Colegio de la Frontera Sur Chetumal Mexico

**Keywords:** Copepods, Mexico, redescriptions, taxonomy, zooplankton

## Abstract

The species of monstrilloid copepods (Monstrilloida: Copepoda: Crustacea) from Mexico that were deposited by the author in the collections of the National Museum of Natural History, Smithsonian Institution in the 1990s are redescribed based on updated descriptive standards. This study includes the re-examination and redescription of type specimens of several species of *Monstrilla* Dana, 1849. The redescriptions were prepared following currently used standards that were set after these species were described. This revision reveals previously unnoticed characters that improve the morphological details of these species; new comparisons of some of these species are also provided. This work will allow a more complete and accurate analysis of these Mexican species.

## ﻿Introduction

Monstrilloids are protelean parasitic copepods infecting marine benthic invertebrates (Huys et al. 2007; Suárez-Morales 2011, 2018; Jeon et al. 2018); the free-living, non-feeding reproductive adults are frequently found during plankton surveys in shallow coastal habitats (Suárez-Morales 2001) and occasionally in oceanic waters (Suárez-Morales and Velázquez-Ornelas 2023, 2024).

The taxonomic study of monstrilloids started with the work by J. D. Dana (1849–1852) and several other studies published during the second half of the 19^th^ century (see Grygier 1995). The taxonomy of this peculiar group is still in development and has been hindered by several problems, including poor original descriptions, and difficulties derived from matching the two sexes in most species, thus promoting a parallel taxonomy for males and females (Suárez-Morales 2011, 2018) and a complex taxonomical story of several species (Suárez-Morales and Grygier 2021). The need of developing descriptive standards for adult monstrilloid copepod comparative work was recognized by Grygier and Ohtsuka (1995); they proposed upgraded standards for describing the monstrilloid body and appendages and also devised a nomenclature to recognize the setal elements and established a unified armature pattern of the adult female monstrilloid antennules for comparative purposes. Subsequently, the same authors (Grygier and Ohtsuka 2008) introduced upgraded standards particularly in reference to cuticular ornamentations and micro-characters. This joint set of descriptive standards has been followed in most of the taxonomical descriptions of monstrilloids published after these contributions. During the 1990’s, I described several species of monstrilloid copepods that were collected from Mexican waters of both the Pacific and Atlantic coasts of Mexico (Suárez-Morales and Gasca-Serrano 1992; Suárez-Morales 1993a, b, 1994; Suárez-Morales and Palomares-García 1995), but was unaware at that time of Grygier and Ohtsuka’s (1995) publication on the upgraded descriptive standards. Overall, this contribution aims to complete, update, and standardize the descriptions of these Mexican species so their morphology is better known and they can be more accurately compared with their congeneric species and identified in zooplankton surveys of Mexican marine waters. Currently, the monstrilloid fauna of Mexico mainly comprises species of the highly diverse genera *Monstrilla* Dana, 1849 and *Cymbasoma* Thompson, 1888, and one species of the monotypic *Spinomonstrilla* Suárez-Morales, 2019. The coastal systems of the Mexican Caribbean and southern Gulf of Mexico are the best surveyed areas in the country, and new species are still being discovered from this subregion (Suárez-Morales 2024) but also in the Gulf of California (Suárez-Morales and Velázquez-Ornelas 2024).

## ﻿Material and methods

Adult monstrilloid copepods were collected during zooplankton surveys from coastal systems of the Mexican Atlantic and Pacific oceans during 1990–1997. The type specimens were deposited in the U.S. National Museum of Natural History, Smithsonian Institution (**USNM**), but some specimens were kept in the Collection of Zooplankton held at El Colegio de la Frontera Sur (**ECOSUR**) in Chetumal, Mexico (ECO-CH-Z), so they were also examined to complete the morphologic details. The species were described in different papers but taxonomically relevant characters like the antennular setation pattern remained undescribed in all cases. This group of species includes members of *Monstrilla* Dana, the most diverse genus in Mexican waters. During the years 2002–2003, I had the opportunity to reexamine these type specimens with the intention of upgrading and completing their morphological descriptions. In all cases, the source zooplankton samples were fixed in formaldehyde solution, and once sorted, monstrilloid copepods were transferred to 70% ethanol for taxonomic examination and long-term preservation. The type specimens were re-examined and illustrated with the aid of a drawing tube adapted to a Carl Zeiss compound microscope. The description of the antennules armature was particularly relevant in this report because details were not provided in the original description of most Mexican species; hence, the armature is fully described herein following Grygier and Ohtsuka’s (1995) nomenclature of the antennule setal elements; therefore, I named each setal element (i.e., spiniform seta, regular seta, aesthetasc) in the illustrations using the alphanumeric labels proposed by Grygier and Ohtsuka (1995) to recognize each element. Consequently, the same labels are used in the descriptive text of each species. The specimens were set in different positions (dorsal, lateral, ventral) to prepare drawings of all taxonomically valuable characters and appendages. The total body length of the specimens examined was measured from the anteriormost end of the cephalothorax (forehead) to the posterior end of the anal somite, excluding the caudal rami. Most type individuals are undissected and deposited in vials with 70% ethanol. The present taxonomic account of the Mexican species of *Monstrilla* follows the general morphological review by Huys and Boxshall (1991) and the upgraded descriptive standards proposed by Grygier and Ohtsuka (1995, 2008) for monstrilloid copepods. Huys et al. (2007) criteria and nomenclature was used to identify the setal armature of the last male antennular segment. In the swimming legs formulae, setae are indicated as Roman numerals and spiniform elements as Arabic numerals.

## ﻿Taxonomy

### ﻿Subclass Copepoda Milne Edwards, 1840

**Order Monstrilloida Sars, 1901**

**Family Monstrillidae Dana, 1849**

**Genus Monstrilla Dana, 1849**

#### ﻿ Monstrilla barbata

Taxon classificationAnimaliaMonstrilloidaMonstrillidae

Suárez-Morales & Gasca-Serrano, 1992

24FEC9BD-C9AB-50F5-A6D2-9BEDB2B0A5D5

Figs 1, 2, 3

##### Type material.

***Holotype*** • adult female, undissected, deposited in the collection of Crustacea, U.S, National Museum of Natural History, Smithsonian Institution. USNM 251756.

##### Type locality.

Bahia de la Ascension, central part of eastern Yucatan Peninsula coast (19°47.00'N, 87°33.20'W). Depth 1.5 m. Date of collection 6 September, 1991.

##### Description of adult female holotype.

**Figure 1.**
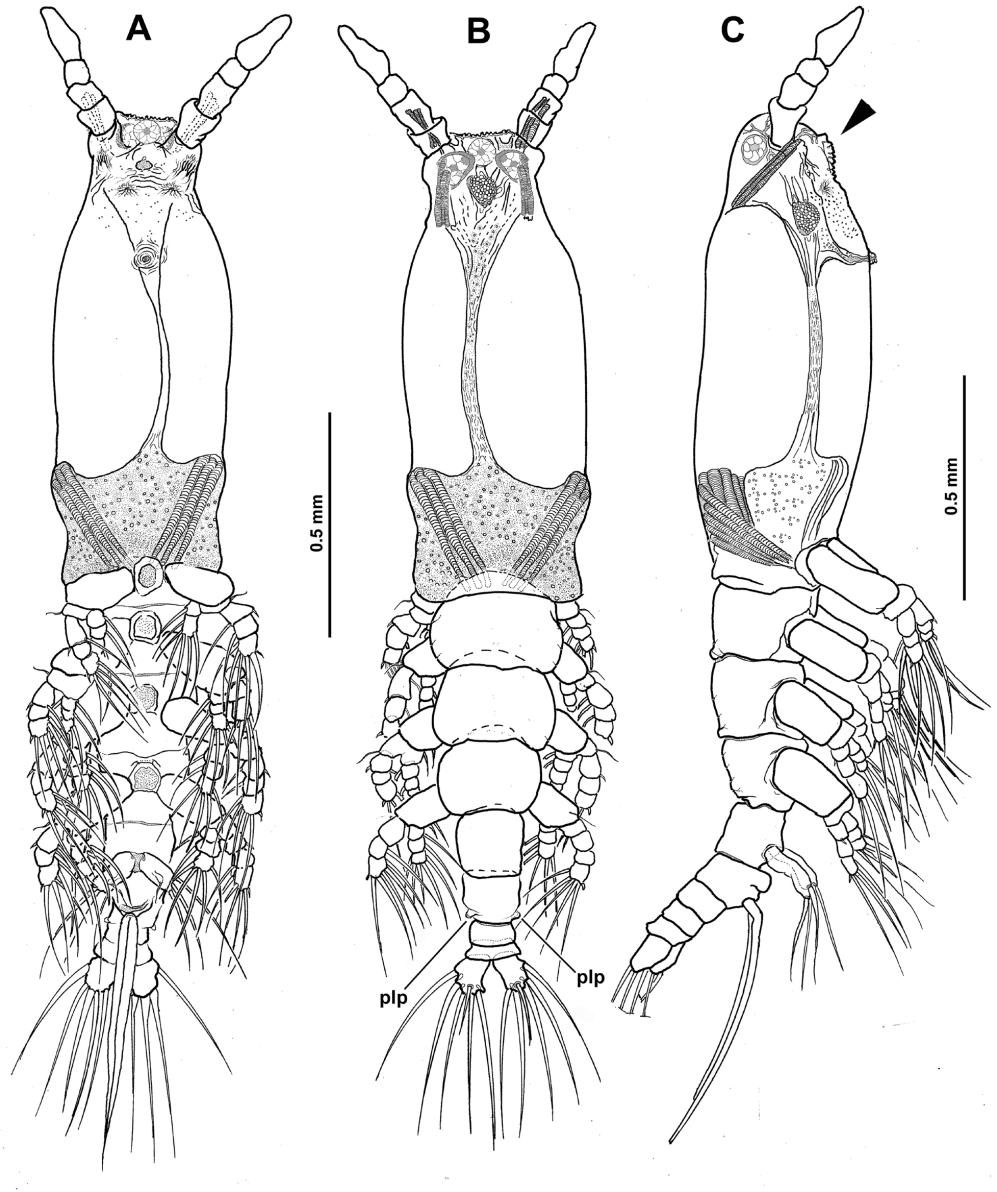
*Monstrilla barbata* Suárez-Morales & Gasca-Serrano, 1992 adult female holotype from the Gulf of California. A. Habitus, ventral; B. Habitus, dorsal; C. Habitus, lateral. Abbreviation: plp = postero-lateral process.

**Figure 2.**
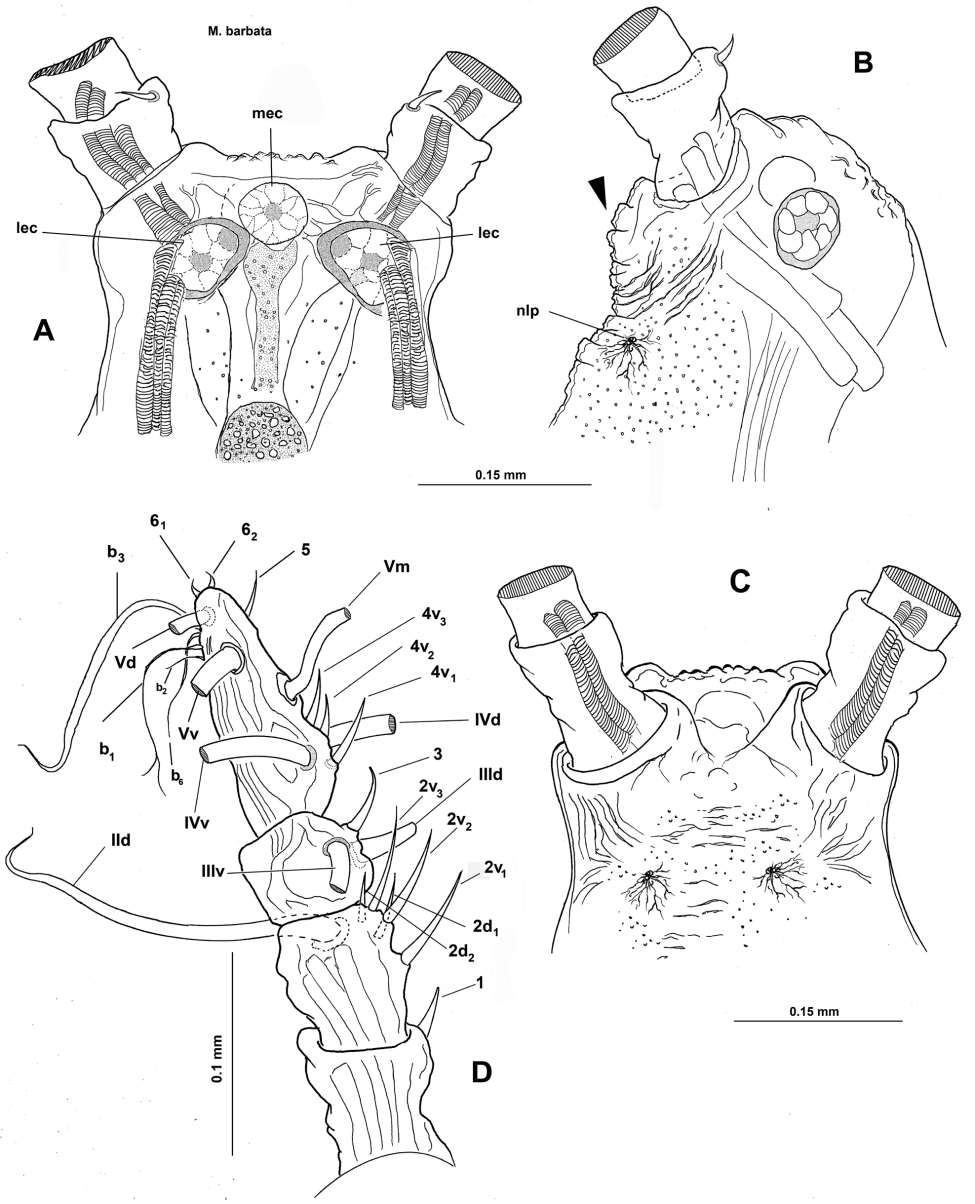
*Monstrilla barbata* Suárez-Morales & Gasca-Serrano, 1992 adult female holotype from the Gulf of California. A. Anterior part of cephalic region; B. Same, lateral (arrowhead indicates medial keel-like process); C. Same, ventral view; D. Left antennule, dorsal. Abbreviations: lec = lateral eye cup; mec = medial eye cup; nlp = nipple-like process.

Total body length measured from forehead to posterior margin of anal somite: 1.82 mm. Cephalothorax 0.947 mm long, representing ~52.1% of total body length. Antennules moderately divergent, representing 16.6% of total body length and 31.5% of cephalothorax length. Oral papilla small, located anteriorly, ~26% posteriorly on ventral surface of cephalothorax (Fig. 1A, C). Eyes represented by relatively small medial cup and two larger, weakly pigmented lateral cups (Fig. 2A, mec, lec). Forehead flat, corrugate, with two large sensilla and field of small cuticular papillae extending to most of cephalic area, including ventral and dorsal surfaces (Fig. 2A, B). Ventral surface of cephalic area bearing: 1) single pair of nipple-like cuticular processes between antennule bases and oral papilla, 2) ventral surface with coarsely corrugate medial keel-like protuberance (Fig. 2B) visible in lateral view of the whole specimen (Fig. 1C, arrowhead); 3) pale pattern of cuticular wrinkles and minute papillae present adjacent to these structures (Fig. 2A).

***Antennules*** (Fig. 2D) 0.34 mm long, almost 17% of total body length; antennules 4-segmented; segments 1–4 clearly divided. Following antennule armature nomenclature by Grygier and Ohtsuka (1995), first segment with short, spiniform element 1; second segment carrying long spiniform elements 2v _1-3_ and 2d_1,2_, dorsal seta IId long; third segment with short, spiniform element 3 and IIIv; elements IIId and IIIv setiform, slender; fourth segment with reduced armature including proximal spiniform element 4d_1_, flexible setae IVd, IV v, and slender ventral aesthetasc 4aes. Fourth segment carrying short spiniform elements 4v_1-3_, setiform elements IVd, IVv, Vm, Vv, Vd, spiniform element 5, three setae of the “b” group inserted on the outer distal margin (b_1_, b_2_, b_3_, b_6_), and reduced apical elements 6_1_ and 6_2_.

**Figure 3.**
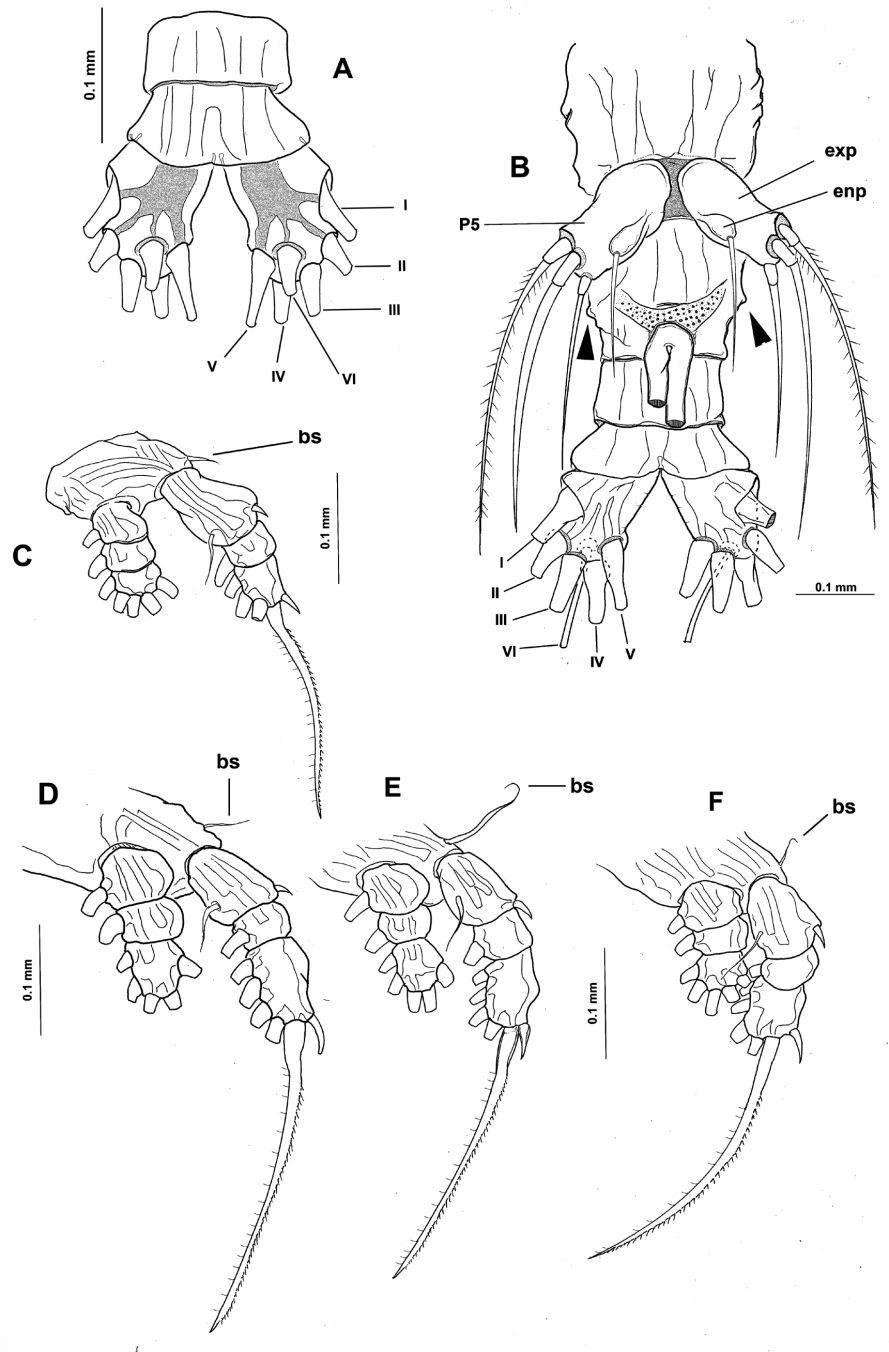
*Monstrilla barbata* Suárez-Morales & Gasca-Serrano, 1992 adult female holotype from the Gulf of California. A. Distal urosomites and caudal rami, dorsal; B. Urosome with fifth legs and ovigerous spines, ventral; C. First swimming leg, anterior; D. Second swimming leg, anterior; E. Third swimming leg, anterior; F. Fourth swimming leg, anterior. Abbreviations: caudal setae I–V sensu Huys and Boxshall 1991; enp= endopod; exp = exopod; P5 = fifth leg.

First pedigerous somite incorporated into cephalothorax; this and succeeding three free pedigerous somites each bearing pair of biramous swimming legs. Pedigerous somites 2–4 together accounting for 26% of total body length in dorsal view. Intercoxal sclerites of legs 1–4 subrectangular, without ornamentation on surface or along distal margin. Basis of legs articulating with rectangular coxa along diagonal line. Basis with thin, simple lateral basipodal seta on legs 1, 2, and 4; on leg 3, this seta thicker, lightly setulate, and 4× longer than on other legs (Fig. 3C–F, bs). Endopods and exopods of swimming legs 1–4 tri-articulate (Fig. 3C–F). Ramus setae all lightly and biserially plumose except for spiniform outer setae on exopodal segments 1 and 3, and inner seta of first exopodal segment, all these being short and slender. Outer apical exopodal seta of swimming legs 1–4 with outer margin smooth, inner margin lightly setose. Armature formula as (not provided in original description):

***Basis Endopod Exopod*** :

leg 1 1-0 0-1; 0-1; 1, 2, 2 I-1; 0-1; I, 2, 2

legs 2–4 1-0 0-1; 0-1; 1, 2, 2 I-1; 0-1; I, 1, 2, 3

***Fifth legs biramous*.** Outer lobe represented by subrectangular exopodal lobe armed with three setae, outermost seta lightly pinnate, being longer, slightly wider than adjacent two exopodal setae; innermost exopodal seta lightly shorter, narrower. Inner endopodal lobe reduced, drop-like, arising proximally from inner margin of exopodal lobe, armed with short, slender apical seta (Fig. 3B).

Urosome consisting of four somites: fifth pedigerous somite, genital double-somite, one free postgenital somite, and short anal somite (Fig. 3B). Genital somite with pair of lateral protuberances on distal 1/2 visible in frontal and ventral views (Fig. 1B, plp; Fig. 3B, arrowheads). Ventral surface of genital somite forming enlarged base of cylindrical shaft with distal genital lappets. Relative length of urosomites, from proximal to distal as: 40: 35: 16.25: 8.76. Caudal rami subquadrate, symmetrical, weakly divergent, ~1.1× longer than wide, each ramus bearing six setae (I–IV), seta VI being shortest (Fig. 3B).

**Male.** Unknown.

##### Remarks.

There are several characters of this species that were not properly described in its original description in 1992 . The holotype habitus was originally provided in dorsal view; I was able to add both ventral and lateral views. The armature of the antennules was largely incomplete and at least one setal element (element 1 sensu Grygier and Ohtsuka 1995) was erroneously illustrated in the original drawings (Suárez-Morales and Gasca-Serrano 1992: fig. 1b). The setal elements present in the holotype specimen are identified, illustrated, and labelled in this redescription, thus allowing a more accurate comparisons among the Caribbean species of *Monstrilla*. Also, the coarse forehead surface noticed in the re-examination was not mentioned or illustrated by Suárez-Morales and Gasca-Serrano (1992); the same is true for the eye cups, which were originally described as present and moderately developed; it was possible now to provide more details about their relative size and pigmentation, recognizing the lateral and medial cups. The cephalic integumental ornamentations were not typically deemed relevant in the taxonomy of monstrilloid copepods, but they can aid to separate species (Cruz Lopes da Rosa et al. 2021). In this redescription of *M. barbata*, I was able to provide new data on the cephalic ventral ornamentation, which includes fields of papillae and wrinkles, a pair of slender sensilla, a pair of nipple-like processes, and details of the medial keel-like corrugate antero-ventral process that is distinctive of the species (Suárez-Morales and Gasca-Serrano 1992). Also, the swimming legs 1–4 were not illustrated in the original description, but drawings of these legs are newly provided in this redescription.

This species was originally compared with *M. longicornis* Thompson, 1890 and *M. lata* Desai & Bal, 1963 based on sharing a bilobed fifth leg carrying four setae. Its fifth leg was also compared with that of *M. reticulata* Davis, 1949. The most distinctive character of *M. barbata* is the beard-like protuberant medial process and was considered unique among species of *Monstrilla* (Suárez-Morales and Gasca-Serrano 1992), but this structure was compared recently with those of other *Monstrilla* species described after *M. barbata*, like *M. pustulata* Suarez-Morales & Dias, 2001 from Brazil, and *M. brevicornis* Isaac, 1974 from Java (see Suárez-Morales and McKinnon 2025). A further comparison of *M. barbata*’s fifth leg with Australian congeneric species was presented by Suárez-Morales and McKinnon (2025). The presence of paired lateral protuberances on the genital double-somite of *M. barbata* was also mentioned by Suárez-Morales and Gasca-Serrano (1992) as another distinctive character of this species and is documented in this paper.

#### ﻿ Monstrilla ciqroi

Taxon classificationAnimaliaMonstrilloidaMonstrillidae

(Suárez-Morales, 1993a)

99F420D6-DA57-5D8B-96C6-448770D8A306

Figs 4, 5

##### Type material.

***Holotype*** • adult female, undissected, deposited in the collection of Crustacea, U.S, National Museum of Natural History, Smithsonian Institution. USNM 251656. Female paratype USNM 251700.

##### Type locality.

Bahía de la Ascensión, Caribbean coast of Mexico (19°47.00'N, 87°33.20'W). Date of collection 5 September 1991.

##### Description of adult female holotype.

**Figure 4.**
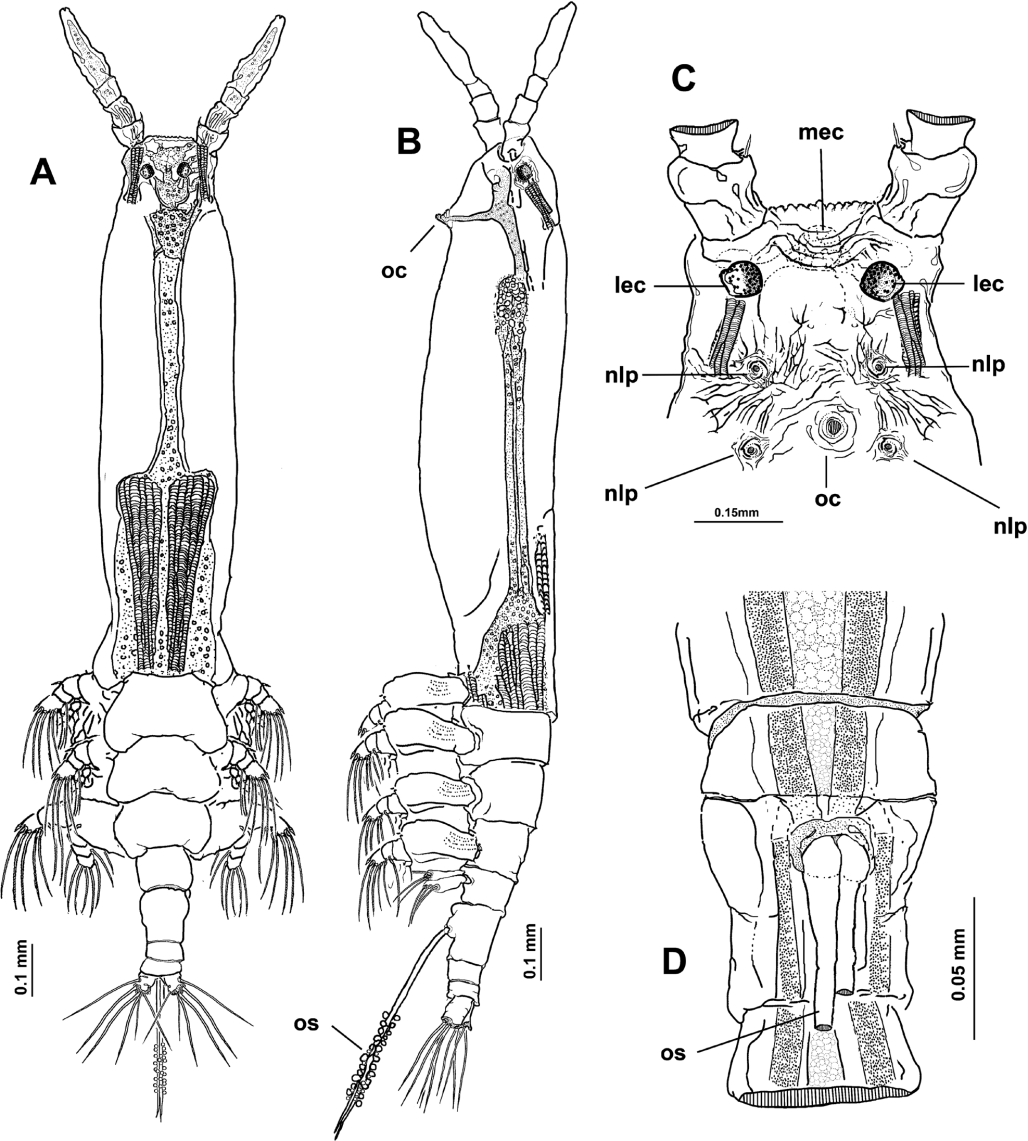
*Monstrilla ciqroi* (Suárez-Morales, 1993a) female holotype from the Mexican Caribbean. A. Habitus, dorsal; B. Habitus, lateral; C. Anterior part of cephalic region; D. Urosome with genital double-somite, ventral. Abbreviations: lec = lateral eye cup; mec = medial eye cup; nlp = nipple-like process; oc = oral cone; os = ovigerous spine.

**Figure 5.**
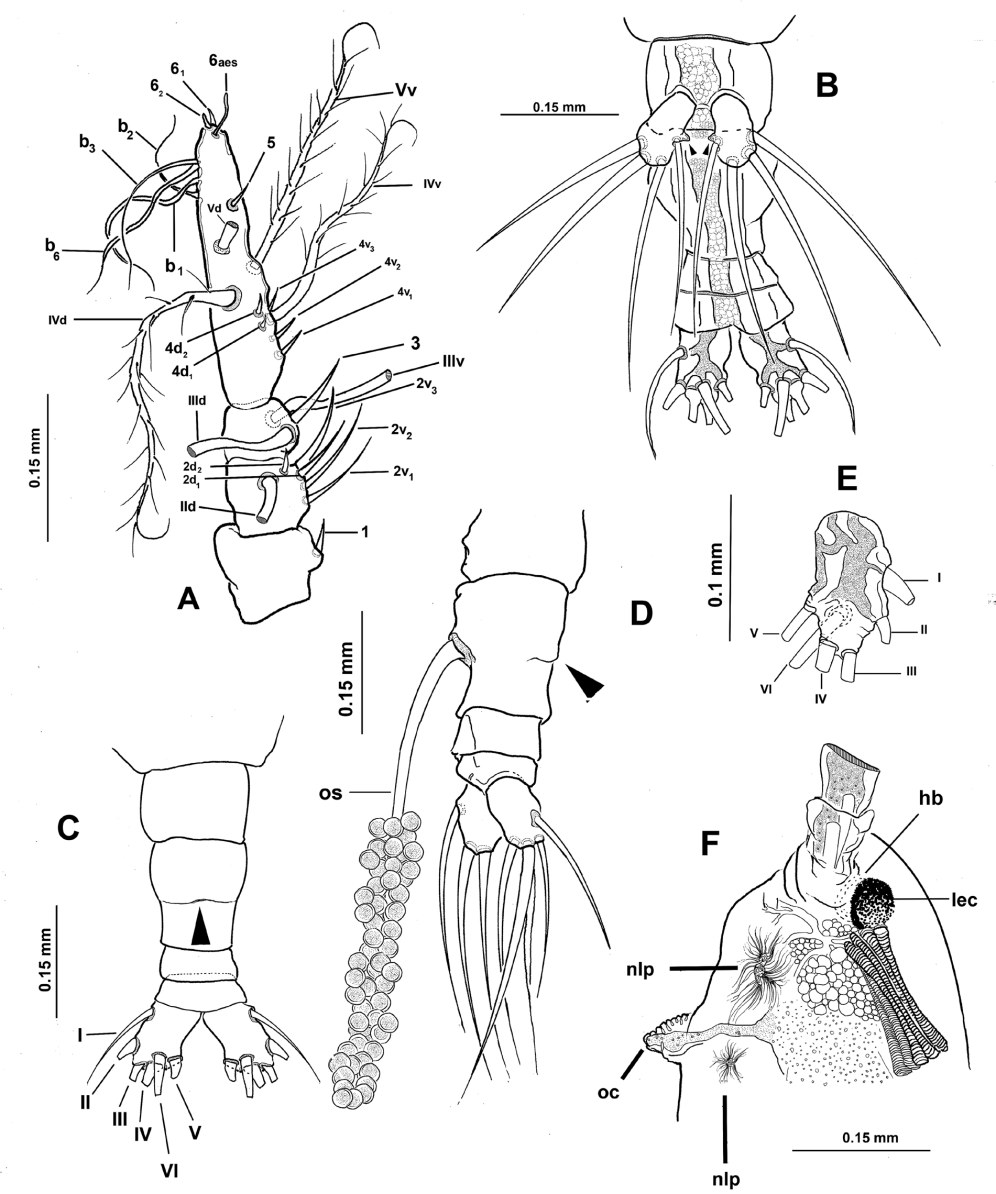
*Monstrilla ciqroi* (Suárez-Morales, 1993a) female holotype from the Mexican Caribbean. A. Left antennule with setal armature (sensu Grygier and Ohtsuka 1995); B. Urosome, ventral; C. Urosome, dorsal (arrowhead indicates dorsal genital suture); D. Urosome, lateral (arrowhead indicates genital suture); E. Caudal ramus; F. Anterior part of cephalic region. Abbreviations: caudal setae I–VI sensu Huys and Boxshall 1991; P5 = fifth leg; nlp = nipple-like process; oc = oral cone; os = ovigerous spine.

Body length of holotype 3.1 mm. Cephalothorax long, cylindrical, with weakly expanded lateral margins (Fig. 4A), cephalothorax representing almost 64% of total body length. Oral cone moderately developed, prominent, papilla-like (Figs 4B, C, 5F, oc), located at 12% of body along ventral surface of cephalothorax (Fig. 4B). Cephalic region with flat ‘forehead’ and weak integumental corrugation, field of transverse integumental wrinkles between antennule bases (Fig. 4C); ventral preoral surface with integumental ornamentation including two pairs of nipple-like processes in both the holotype and paratype specimens (Figs 4C, 5F, nlp) and medial low protuberance (in Fig. 5F, nlp). Eyes comprising two lateral cups and medial cup (Fig. 4C, lec, mec); lateral eye cups strongly pigmented, with a diameter ~0.6× as that of larger, weakly pigmented medial eye cup (Figs 4C, 5F); small hyaline bodies (sensu Suárez-Morales 2018) visible anteriorly to lateral eye cups (Figs 4C, 5F, dotted lines).

***Urosome consisting of four somites***: fifth pedigerous somite (with fifth legs), genital double-somite ventrally carrying paired ovigerous spines barely reaching beyond distal end of caudal rami and attached egg cluster (Fig. 5D), free preanal somite, and anal somite carrying pair of caudal rami; length ratio of urosomites (from proximal to distal) 28.3: 48.3: 13.3: 10.1 (Fig. 5D). Genital double-somite with weakly expanded lateral margins on proximal 1/2 (Fig. 4D), with incomplete transverse suture visible in dorsal and lateral view (Fig. 5C, D arrowheads); pair of slender ovigerous spines on ventral surface, carrying eggs mass (Figs 4B, 5D, os). Caudal rami subrectangular, ~1.5× as long as broad, each armed with six caudal setae (I–VI), seta VI being shortest (Fig. 5B, E).

***Antennules*** 0.64 mm in length, representing ~21% of total body length and almost 32% of cephalothorax length (Fig. 4A, B); as usual in female monstrilloids, antennules distinctly 4-segmented, anteriorly directed, weakly divergent (Fig. 4A); segments 1–3 divided, segments 3 and 4 partly fused (Fig. 5A); length ratio of antennular segments (proximal to distal) 16.5: 14.3: 12.1: 57.1 (Fig. 5A). Following Grygier and Ohtsuka’s (1995) setal nomenclature for female antennules, first segment with reduced, slender setal element 1, second segment bearing setiform element IId, and long, curved spiniform elements 2v_1-3_, 2d_1,2_,; third segment with spiniform smooth element 3 and adjacent setiform elements IIId and IIIv, fourth segment longest of antennule, separated from third by deep intersegmental suture, proximal 1/2 armed with short spiniform elements 4v_1-3_, 4d_1,2_, long, biserially setulated setiform elements IVd and IVv; distal 1/2 armed with setiform elements Vv (biserially setulated), Vd, short spiniform element 5, and several setae of the “b-group” (b_1_, b_2_, b_-3_, b_6_) on outer distal margin; apical elements 6_1_, 6_2_, and 6aes reduced (Fig. 5A).

First pedigerous somite and succeeding three free thoracic somites each bearing well-developed pair of biramous swimming legs (Fig. 4A, B), all with exopodite longer than endopodite. Setal armature pattern as in *M. barbata* (this document). Armature of swimming legs 1–4 as:

***Legs Basis Endopod Exopod*** :

Leg 1 1-0 0-1; 0-1;1, 2, 2 I-1; 0-1; I, 2, 2

Legs 2–4 1-0 0-1; 0-1; 1, 2, 2 I-1; 0-1; I, 2, 3

***Fifth legs*** (Fig. 5B) represented by a single oblong exopodal lobe armed with four setae, three setae inserted terminally, subequal in length and breadth; fourth seta representing the endopod inserted laterally on midlength of inner margin adjacent to small inner beak-like protuberance (Fig. 5B, arrowheads).

##### Remarks.

This species was originally assigned to *Monstrillopsis* Sars, 1921 based on the well-developed eyes and the forward location of the oral cone (Suárez-Morales 1993a). Affinities with the invalid genus *Strilloma* Isaac, 1975 were also suggested in its original description (Suárez-Morales and Gasca 2004). It was compared therein with other species of *Monstrillopsis* recognized by Isaac (1975) like *M. angustipes* Isaac, 1974, *M. dubia* (T. Scott, 1904), *M. gracilis* (Gurney, 1927), and *M. reticulata* Davis, 1949. The main character used to separate *M. ciqroi* from species of *Monstrillopsis* was the fifth leg structure and armature; *M. reticulata* fifth leg was deemed morphologically closest, but differences in the antennule armature were also mentioned to distinguish these two species. Further research (Suárez-Morales and Gasca 2004) confirmed *Strilloma* as invalid, and the first taxonomic revision of *Monstrillopsis* by Suárez-Morales et al. (2006) resulted in the inclusion of both *Monstrillopsis reticulata* and *M. ciqroi* as members of *Monstrilla*.

*Monstrilla ciqroi* has relevant affinities with other Caribbean species sharing a fifth leg with the same setal armature of the fifth legs (3 exopodal, 1 endopodal) like *M. rebis* Suárez-Morales, 1993, *M. barbata*, and *M. xcalakensis* Suárez-Morales, 2024, but *M. ciqroi* diverges in the size and structure of the endopodal lobe. In both *M. rebis* and *M. barbata* (see Fig. 3B) the inner lobe is a relatively well defined, armed with a distal seta and in *M. xcalakensis* a strongly developed endopodal lobe is present, approximately the same size of the exopodal lobe (Suárez-Morales, 2024: fig. 3A). In *M. ciqroi* the fifth leg inner seta likely represents the endopod, lacking a structured endopodal lobe. The number of caudal setae has been a relatively strong character related in the definition of some monstrilloid genera: three setae in *Cymbasoma* (Suárez-Morales and McKinnon, 2016), four setae in *Monstrillopsis* (Suárez-Morales et al. 2006), and five or six setae in *Monstrilla* and *Caromiobenella* (Jeon et al. 2018; Suárez-Morales and McKinnon 2025). In the original description of *M. ciqroi*, only five caudal setae were observed; this re-examination of the holotype specimen allowed me to determine that the actual number of caudal setae is six (Fig. 5B, C, E); this finding supports the decision of including *M. ciqroi* as a member of *Monstrilla*. Overall, this species can be distinguished from other Mexican Caribbean species of *Monstrilla* by its strongly pigmented eyes, fifth leg armed with four setae, and the lack of a structured endopodal lobe.

#### ﻿ Monstrilla rebis

Taxon classificationAnimaliaMonstrilloidaMonstrillidae

Suárez-Morales, 1993a

09B7B0D7-9AE9-50FA-8789-A1FD4F303EC8

Figs 6, 7

##### Type material.

***Holotype*** • adult female, undissected, deposited in the collection of Crustacea, U.S, National Museum of Natural History, Smithsonian Institution. USNM 251655. Adult female paratype ECO-CHZ-00067.

##### Type locality.

Bahía de la Ascensión, Caribbean coast of Mexico) (19°45.09'N, 87°30.00'W). Date of collection 4 September 1991.

##### Description of adult female holotype.

**Figure 6.**
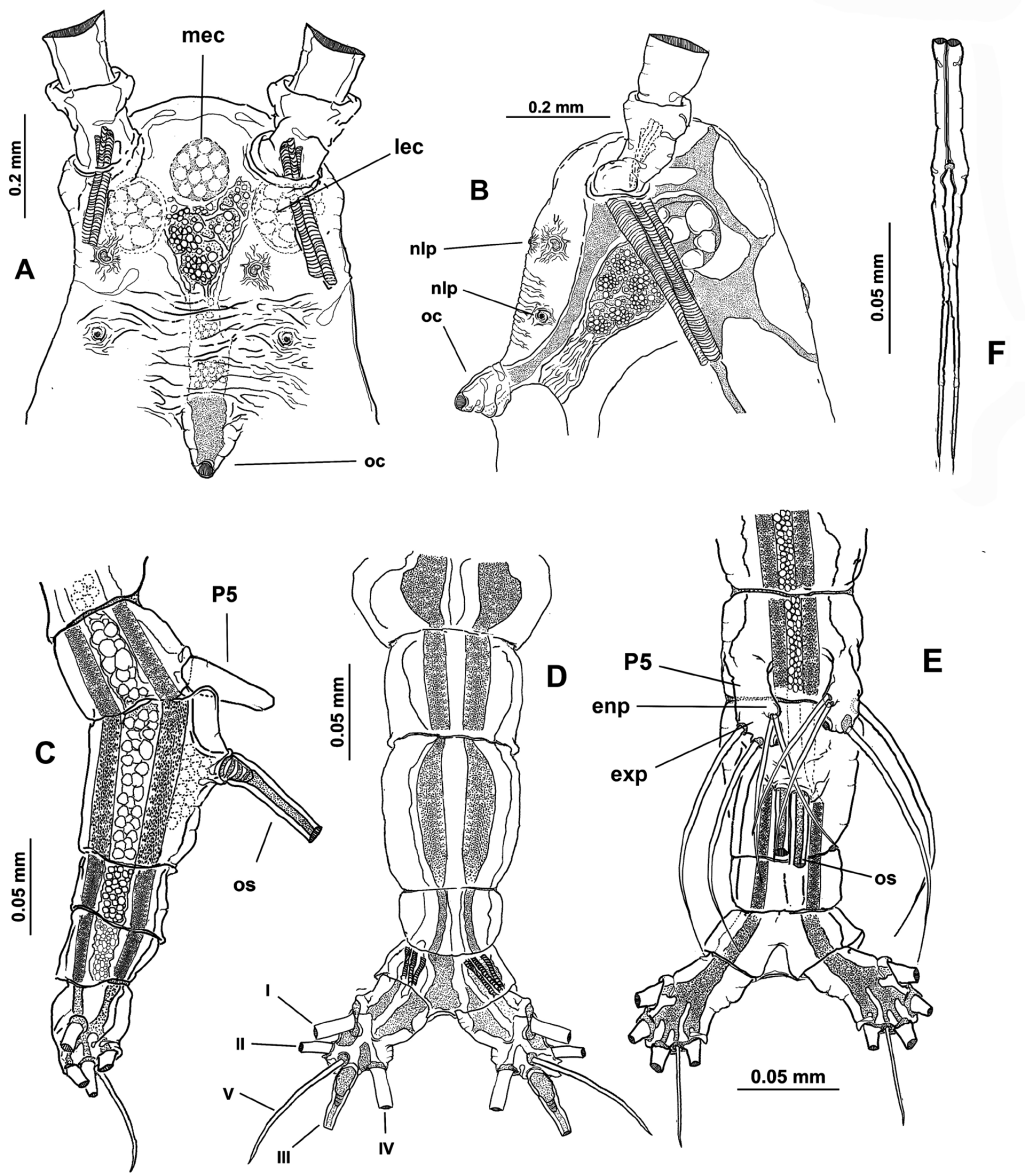
*Monstrilla rebis* (Suárez-Morales, 1993a) female holotype from the Mexican Caribbean. A. Anterior part of cephalic region, ventral; B. Same, lateral; C. urosome, lateral; D. Urosome, lateral; E. Urosome, dorsal; F. Ovigerous spines, distal points. Abbreviations: enp = endopodal lobe; exp = exopodal lobe; lec = lateral eye cup; mec = medial eye cup; nlp = nipple-like process; oc = oral cone; os = ovigerous spine; P5 = fifth leg.

Body length of holotype 2.98 mm, paratype body length 2.82 mm. Cephalothorax cylindrical with weakly expanded lateral margins, relatively short, representing almost 55% of total body length. Oral cone well developed, prominent, papilla-like (Fig. 6B, oc), located 22% of way back along ventral surface of cephalothorax. Cephalic region anteriorly expanded in dorsal view, ‘forehead’ rounded, smooth, weakly produced (Fig. 6A); ventral preoral surface with integumental ornamentation including two pairs of nipple-like processes with adjacent integumental wrinkles in both the holotype and paratype (Fig. 6B, nlp). Eyes comprising two relatively large lateral cups (Fig. 6A, lec) and ventral medial cup (Fig. 6A, mec), latter smaller than lateral cups; lateral eye cups weakly pigmented, with a diameter ~1.2× as that of smaller unpigmented medial eye cup (Fig. 6A).

***Urosome consisting of four somites***: fifth pedigerous somite (carrying fifth legs), genital double-somite with pair of ovigerous spines reaching well beyond distal end of caudal rami (Suárez-Morales 1993a: fig. 1a), free preanal somite, and anal somite carrying pair of caudal rami; length ratio of urosomites (from proximal to distal) 17.5: 23.5: 33.25: 12.5: 13.25 (Fig. 6C, D). Genital double-somite longest of urosome, with weakly expanded lateral margins (Fig. 6D, E); pair of slender ovigerous spines on ventral surface; spines equally long, both ending in thin, straight parallel points (Fig. 6F). Caudal rami subrectangular (Fig. 6D), ~1.4× as long as broad, each armed with five caudal setae (I–V), seta V being shortest (Fig. 6D).

**Figure 7.**
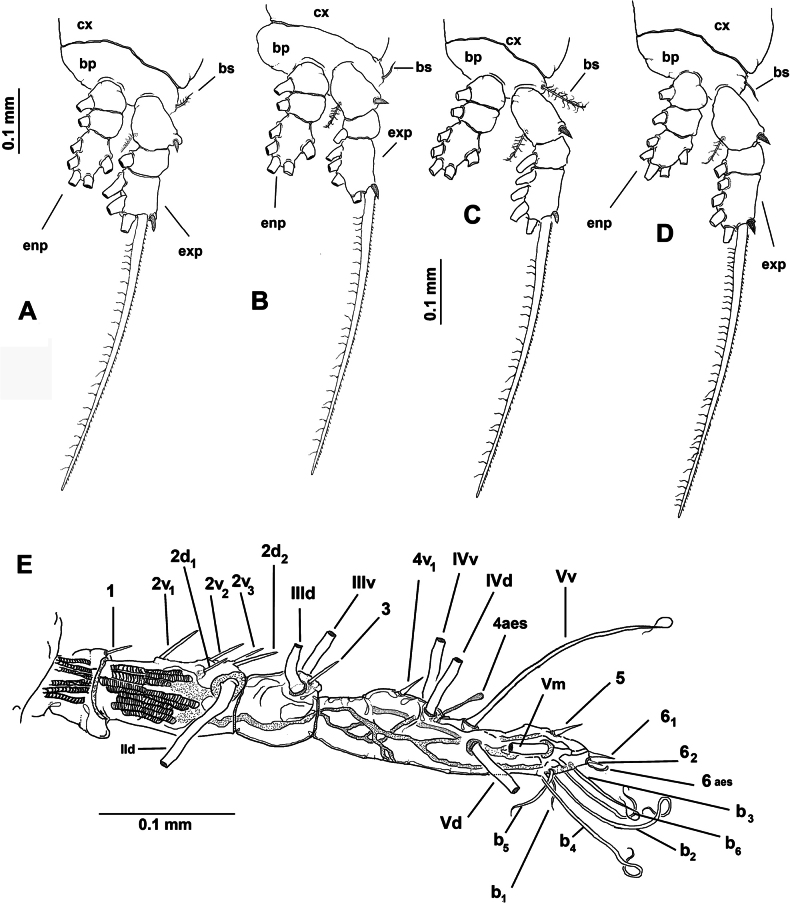
*Monstrilla rebis* (Suárez-Morales, 1993a) female holotype from the Mexican Caribbean. A. First swimming leg, anterior; B. Second swimming leg, anterior; C. Third swimming leg, anterior; D. Fourth swimming leg, anterior; E. Right antennule, dorsal with setal armature sensu Grygier and Ohtsuka (1995). Abbreviations: bp = basipod; bs = basipodal seta; cx = coxa; enp = endopod; exp = exopod.

***Antennules*** 0.64 mm in length, representing ~23% of total body length and almost 44% of cephalothorax length (Suárez-Morales 1993a: fig. 1a); as usual in female monstrilloids, antennules distinctly 4-segmented, relatively slender, anteriorly directed, weakly divergent; intersegmental divisions segments 1–3 complete (Fig. 7E); length ratio of antennular segments (proximal to distal) 10.71: 25.71: 14.28: 49.3 (Fig. 7E). Following Grygier and Ohtsuka’s (1995) setal nomenclature for female antennules, first segment with reduced, slender setal element 1, second segment bearing setiform element IId, and stout, slender spiniform elements 2v_1-3_ and 2d_1,2_; third segment with slender, stout spiniform element 3 and adjacent setiform elements IIId and IIIv, fourth segment longest of antennule, proximal 1/2 armed with short spiniform elements 4v_1_, setiform elements IVv and IVd, and short aesthetasc 4 aes. Distal 1/2 of fourth segment with long, biserially setulated setiform elements IVd and IVv, Vm, and Vv plus short spiniform element 5; outer distal margin carrying several unbranched, slender setae of the “b-group” (b_1-6_); apical elements 6_1,2_ spiniform, acute, with reduced adjacent aesthetasc 6aes (Fig. 7E).

First pedigerous somite and succeeding three free thoracic somites each bearing well-developed pair of biramous swimming legs (Suárez-Morales 1993a: fig. 2b, c, d), all with exopodite longer than endopodite. Swimming legs 1–4 slender, with setal armature pattern as shown in Fig. 7A–D. Armature formula of swimming legs 1–4 as:

***Legs Basis Endopod Exopod*** :

Leg 1 1-0 0-1; 0-1; 1-2-2 I-1; 0-1; I, 2, 2

Legs 2–4 1-0 0-1; 0-1; 1-2-2 I-1; 0-1; I, 2, 3

***Fifth legs*** (Fig. 6E, P5) biramous, represented by long, subrectangular exopodal segment armed with two equally long terminal setae; inner (endopodal) lobe reduced, comprising small inner protuberance armed with two apical setae. Fifth leg setae long, reaching beyond distal margin of anal somite (Fig. 6E).

**Male.** Unknown.

##### Remarks.

In the original description, the position of the oral cone (at the anterior ¼ of the cephalothorax) was proposed as the main distinctive character of this species (Suárez-Morales 1993a); this remark was based on Isaac’s (1975) definition of *Monstrilla*, which, in reference to this particular character, is misleading; however, at that time it was one of the most used sources on monstrilloid morphology. In *M. rebis*, the oral cone was originally described as located at 15% of the body on the cephalothorax ventral surface, but in fact it is positioned at 22–25% in both the holotype and the paratype. The antennule length < ½ cephalothorax length (44%) was considered as the second most relevant character to separate *M. rebis* from its known congeneric species; it was compared with monstrilloid species having clearly longer antennules, like *M. conjunctiva* Giesbrecht, 1902 and *C. helgolandica*Jeon et al. 2018. The third distinctive character mentioned was the structure and armature of the fifth legs, showing a unique pattern of two exopodal, two endopodal setae. It was compared with other species of *Monstrilla* with a total of four setae on the fifth leg arranged in a different combination (i.e., 3 exopodal, 1 endopodal), such as *M. wandelii* Stephensen, 1913, *C. helgolandica* (Claus, 1863), and *M. conjunctiva* Giesbrecht, 1902. Other species of *Monstrilla* with four setae (3, 1) on the female fifth leg include *M. longiremis* Giesbrecht, 1893, *M. longicornis* Thompson, 1890, and related species.

Most importantly, there is only another species of *Monstrilla* sharing the same fifth leg setation pattern with *M. rebis* (two endopodal, two exopodal setae), the Philippine *M. grygieri* Suárez-Morales, 2000, from which it can be distinguished by several characters, including the length and segmentation of the antennules: in *M. grygieri* the antennules are clearly longer (73% of cephalothorax length) than in *M. rebis* (40%) and its segments 2–4 are partly fused (Suárez-Morales 2000: fig. 2A, B) vs completely divided antennular segments 1–4 in *M. rebis*; the position of the oral cone clearly diverges in these two species, this structure is located approximately halfway along the cephalothorax ventral surface in *M. grygieri* (Suárez-Morales 2000: fig. 1A) vs the anterior ¼ of the cephalothorax in *M. rebis*; and the number of caudal setae, six in *M. grygieri* (Suárez-Morales 2000: fig. 2D), vs five in *M. rebis*.

#### ﻿ Monstrilla reidae

Taxon classificationAnimaliaMonstrilloidaMonstrillidae

Suárez-Morales, 1993b

F23DF23D-6094-593B-BD51-F9654F62AC81

Figs 8, 9, 10, 11

##### Type material.

***Holotype*** • male, undissected, deposited in the U.S. National Museum of Natural History (USNM-251698). Paratype adult male, undissected. Specimens preserved in 70% ethanol, vial USNM-251699.

##### Type locality.

Bahia de la Ascension, central part of the eastern coast of the Yucatan Peninsula (19°45.09'N, 87°30.00'W), Caribbean coast of Mexico.

##### Description of adult male holotype.

**Figure 8.**
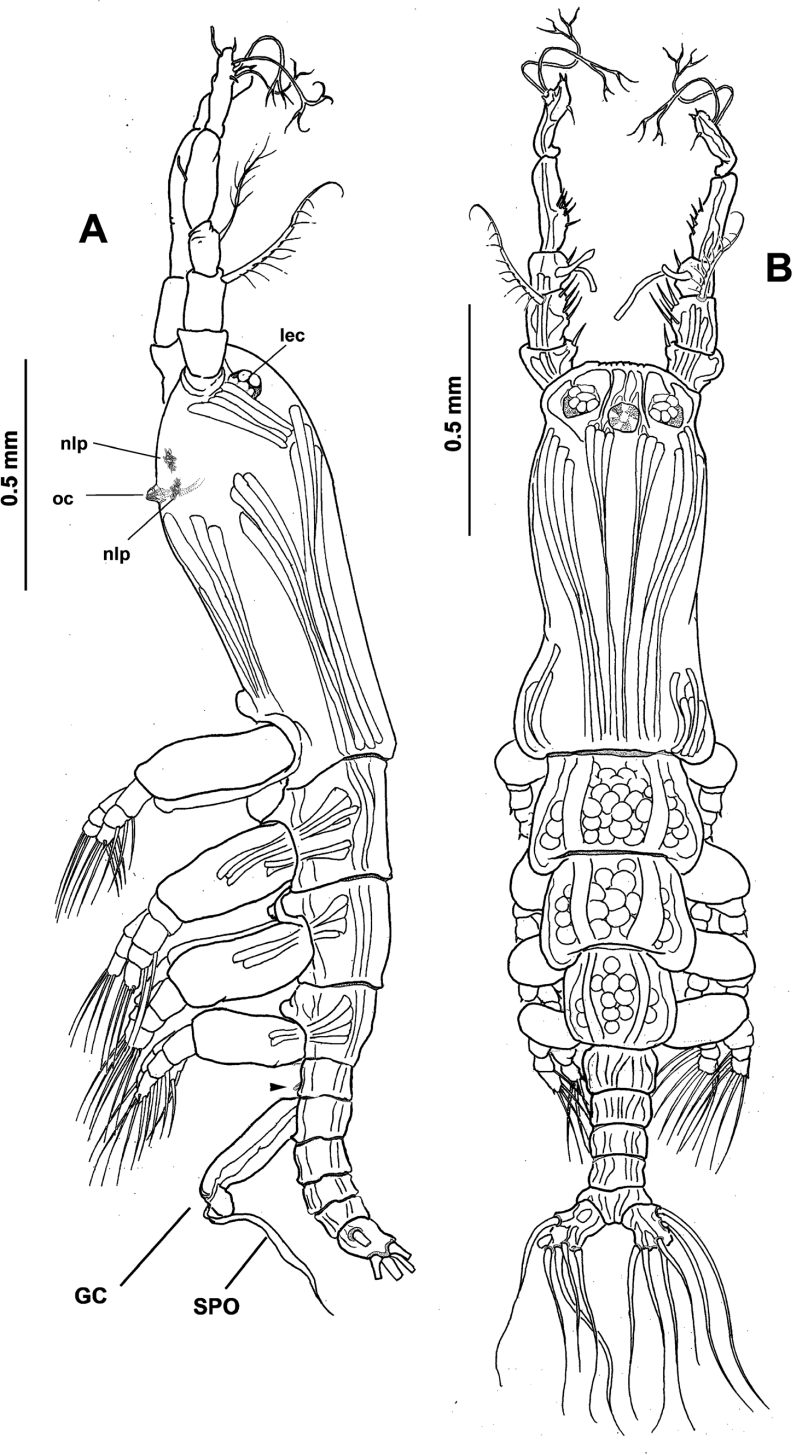
*Monstrilla reidae* Suárez-Morales, 1993 male holotype from the Mexican Caribbean. A. Habitus, lateral (arrowhead indicates fifth leg bud); B. Habitus, dorsal. Abbreviation: oc = oral cone.

**Figure 9.**
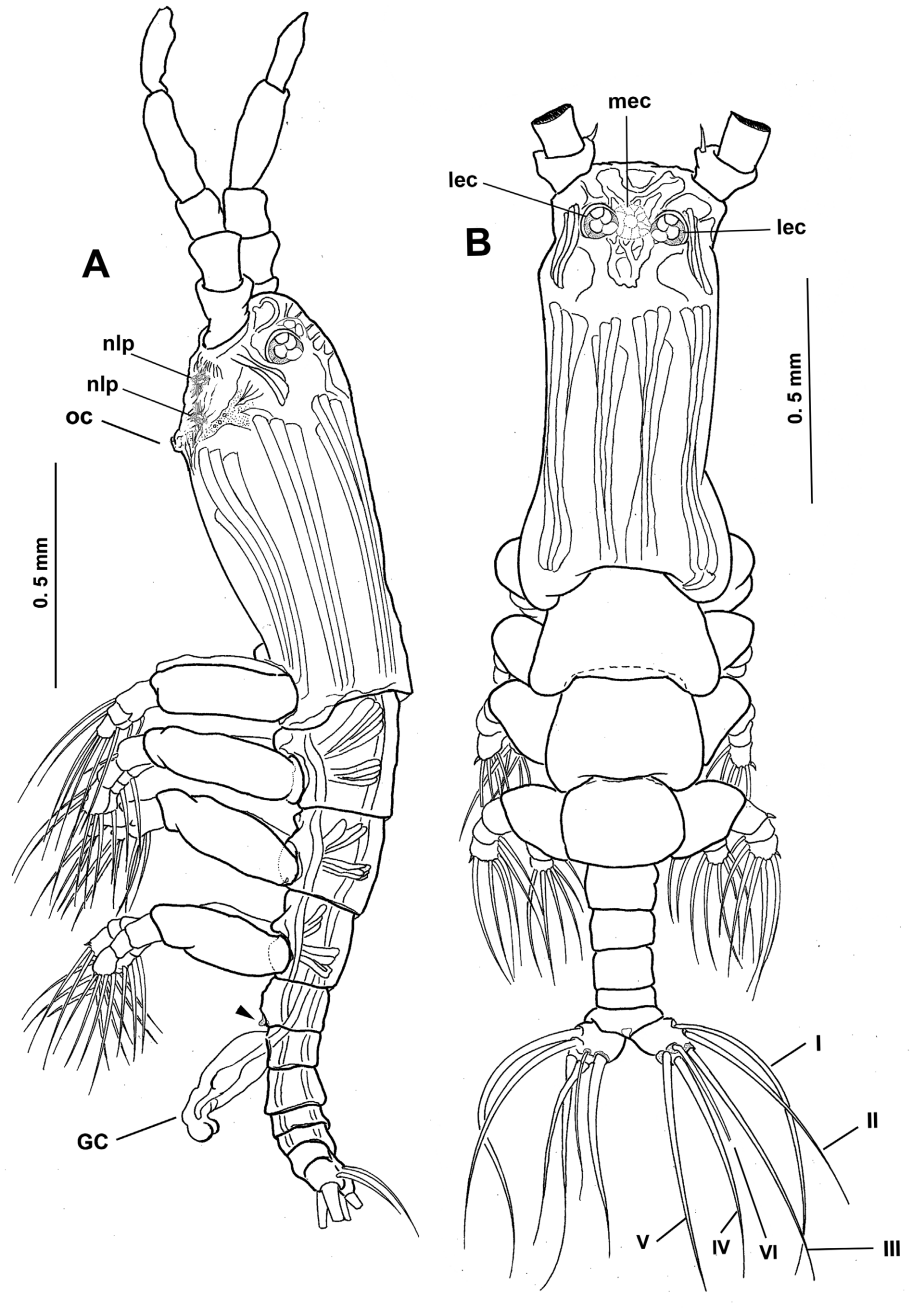
*Monstrilla reidae* Suárez-Morales, 1993 male paratype from the Mexican Caribbean. A. Habitus, lateral (arrowhead indicates fifth leg bud); B. Habitus, dorsal.

**Figure 10.**
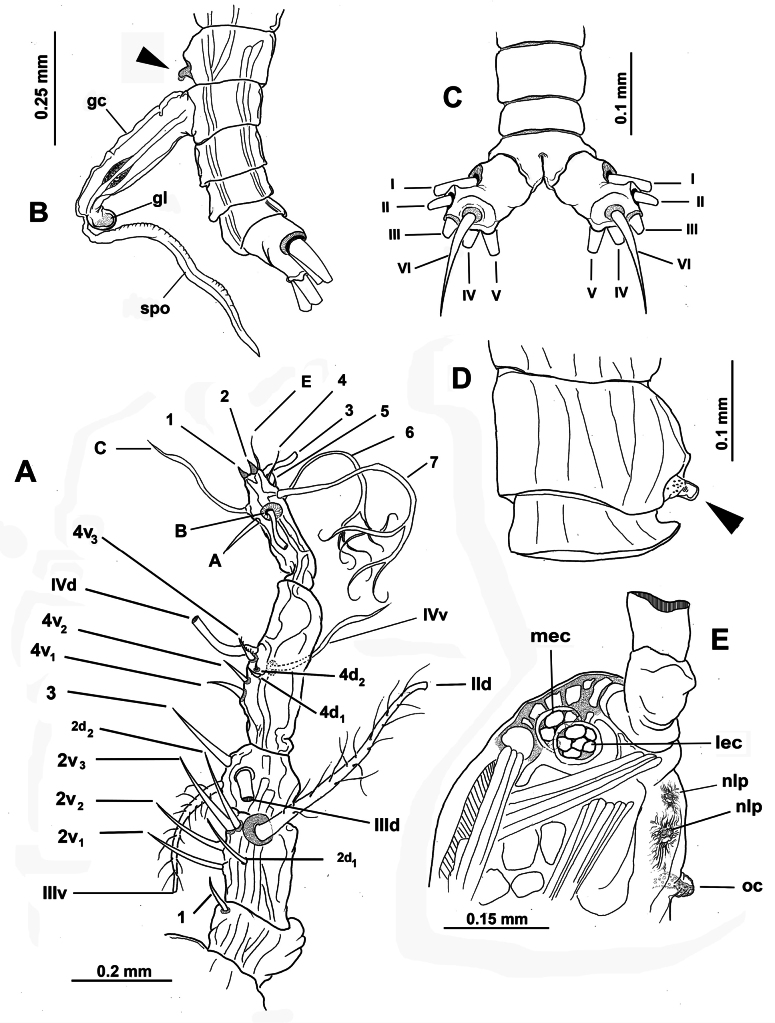
*Monstrilla reidae* Suárez-Morales, 1993 male holotype from the Mexican Caribbean. A. Right antennule, dorsal with setal armature sensu Grygier and Ohtsuka 1995; B. Urosome, lateral (arrowhead indicates fifth leg bud); C. Urosome with caudal rami, dorsal; D. Fifth pedigerous somite, lateral (arrowhead indicates fifth leg bud); E. Anterior part of cephalic region, lateral. Abbreviations: caudal setae I–VI sensu Huys and Boxshall 1991; gc = genital complex; gl = genital lappet; lec = lateral eye cup; mec = medial eye cup; nlp = nipple-like process; oc = oral cone; spo = everted spermatophore.

**Figure 11.**
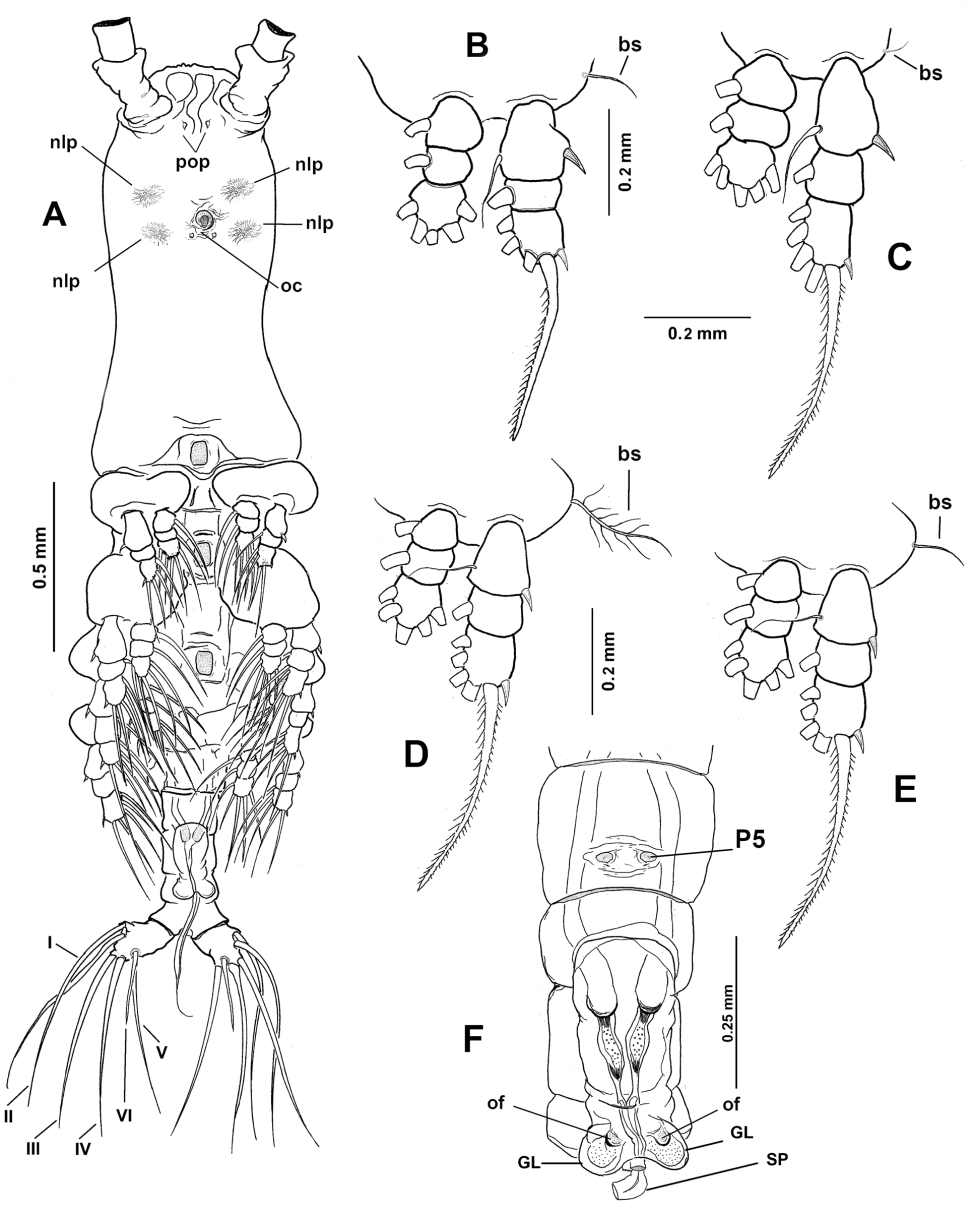
*Monstrilla reidae* Suárez-Morales, 1993 male holotype from the Mexican Caribbean. A. Habitus, ventral; B. First swimming leg, anterior; C. Second swimming leg, anterior; D. Third swimming leg, anterior; E. Fourth swimming leg, anterior; F. Urosome with genitalia, ventral. Abbreviations: bs = basipodal seta; gl = genital lappets; of = opercular flaps; P5 = fifth leg buds; nlp = nipple-like processes; pop = preoral pores; oc = oral cone; sp = spermatophore.

Total body length of holotype 2.3 mm (Fig. 8), length of paratype 2.4 mm (Fig. 9). Cephalothorax robust, representing 48.3% of body (Fig. 8A, B). Oral cone moderately developed, located at 28% from anterior margin of cephalothorax ventral surface in both the holotype and paratype specimens (Figs 8A, 9A, 10E, oc). Eyes weakly pigmented, represented by two lateral eye cups and medial eye cup at anterior part of cephalothorax, medial and lateral cups ~the same diameter in both the holotype (Fig. 10E; mec, lec) and paratype (Fig. 9B). Cephalothorax integumental ornamentation including pair of medial preoral pores (Fig. 11A, pop), two pairs of nipple-like processes with adjacent field of wrinkles (Figs 10E, 11A, nlp).

Urosome relatively slender (Fig. 10B), almost 21% of total body length; urosome comprising fifth pedigerous somite, genital somite, one free somite, preanal and anal somites, the latter holding pair of caudal rami; relative length of urosomites, from proximal to distal: 26.66: 22.22: 24.44: 15.55: 11.11. Fifth pedigerous somite with straight lateral margins, ventral surface moderately produced, carrying pair of reduced fifth leg buds (Figs 8A, 9A, 10D, arrowheads). Succeeding genital somite carrying genital apparatus. Free postgenital somite subquadrate, with smooth ventral and lateral surfaces. Preanal and anal somites subequally long, with smooth lateral and dorsal surfaces; anal somite carrying caudal rami. Genital complex arising ventrally on genital somite (Figs 8A, 9A, 10B, GC), apparatus comprising long cylindrical shaft with smooth lateral margins; shaft distally branching into pair of short, divergent rounded genital lappets (Figs 10B, 11F, GL); lappets medially conjoined by rounded concave process with medial genital opening with everted spermatophore (Figs 9A, 10B, 11F, SPO). Caudal rami subrectangular, 1.6× as long as wide (Fig. 10C); rami armed with six caudal setae subequal in length and width; seta VI relatively shorter (Figs 8B, 10C, 11A).

As usual in male *Monstrilla*, antennules 5-segmented, geniculate. Ratio of length of the antennule segments, from proximal to distal: 7.79: 15.58: 9.74: 24.67: 18.83. Fourth segment is the longest (Fig. 10A). Antennules ~35% of total body length and 75% of cephalothorax length. Following nomenclature by Grygier and Ohtsuka (1995) for antennular segments 1–4, first segment with short, spiniform element 1; second segment carrying slender spiniform elements 2d_1,2_ and 2v_1,3_ and relatively short, lightly setulated dorsal seta IId; third segment with long, stout, spiniform element 3 and lightly setulated setiform elements IIIv and IIId; fourth segment comprising proximal elements 4d_1,2_ and 4v_1-3_, and lightly setulated setiform elements IVv and IVd; distal 1/2 of segment with straight lateral margins, armature absent. Following Huys et al. (2007) nomenclature for the setation of the male antennular fifth segment, outer margin with slender elements 3–7, the last two (6 and 7) dichotomously branched. Inner margin armed with slender, unbranched elements A, B, and C. Short spiniform elements 1 and 2 and aesthetasc E present in apical position (Fig. 10A).

Incorporated first pedigerous somite and succeeding three pedigerous somites each bearing a pair of well-developed swimming legs, subequal in length. Basis of four swimming legs armed with single basipodal seta inserted on outer margin (Fig. 11B–E, bs); seta on leg 3 lightly setulated, longer and thicker than in other legs (Fig. 11D). Endopods and exopods of swimming legs1–4 each with three segments. Armament formula of swimming legs as:

***Legs Basis Endopod Exopod*** :

Leg 1 1-0 0-1; 0-1; 1, 2, 2 I-1; 0-1; I, 2, 2

Legs 2–4 1-0 0-1; 0-1; 1, 2, 2 I-1; 0-1; I, 2, 3

***Fifth legs*** reduced, represented by pair of buds arising ventrally from fifth pedigerous somite. Urosome consisting of fifth pedigerous, genital double and two free abdominal somites, length ratio of anterior to posterior segments being 30.9:24.6: 16.5: 18.2:9.8.

**Female.** Unknown.

##### Remarks.

In the original description (Suárez-Morales 1993b: 718) of this species the armature formula of the swimming legs 1–4 was incorrect, failing to show that the exopodal rami setation pattern differs between the first leg (with one seta less on the third segment, 5 in total) and that of the other swimming legs (with 6 setae on the third segment). This redescription has allowed me to correct this mistake. In addition, the reduced fifth legs buds arising from the ventral surface of the fifth pedigerous somite was unnoticed by Suárez-Morales (1993b) and not included in the original description. The presence of a reduced fifth leg is frequent in male *Monstrilla*. Its development is variable, and the Caribbean *M. mahahualensis* and related species likely have the strongest fifth leg development within the genus *Monstrilla*. Contrastingly, fifth legs are absent in the closely related genus *Caromiobenella* (Jeon et al. 2018), with the exception of *C. brasiliensis* (Cruz Lopes da Rosa et al. 2021).

#### ﻿ Monstrilla elongata

Taxon classificationAnimaliaMonstrilloidaMonstrillidae

Suárez-Morales, 1994

4286AAE3-DAF3-50BE-8184-A33BC127EF32

Figs 12, 13, 14, 15

##### Type material.

***Holotype*** • adult female, undissected, ethanol-preserved, vial deposited in the collection of Crustacea, U.S. National Museum of Natural History, Smithsonian Institution, USNM 259488.

##### Type locality.

Puerto Escondido coastal system, eastern coast of the Baja California Peninsula Puerto Morelos, northern part of the Mexican Caribbean coast (20°51.40'N, 86°54.15'W). Date of collection 18 November 1993.

##### Description of adult female holotype.

**Figure 12.**
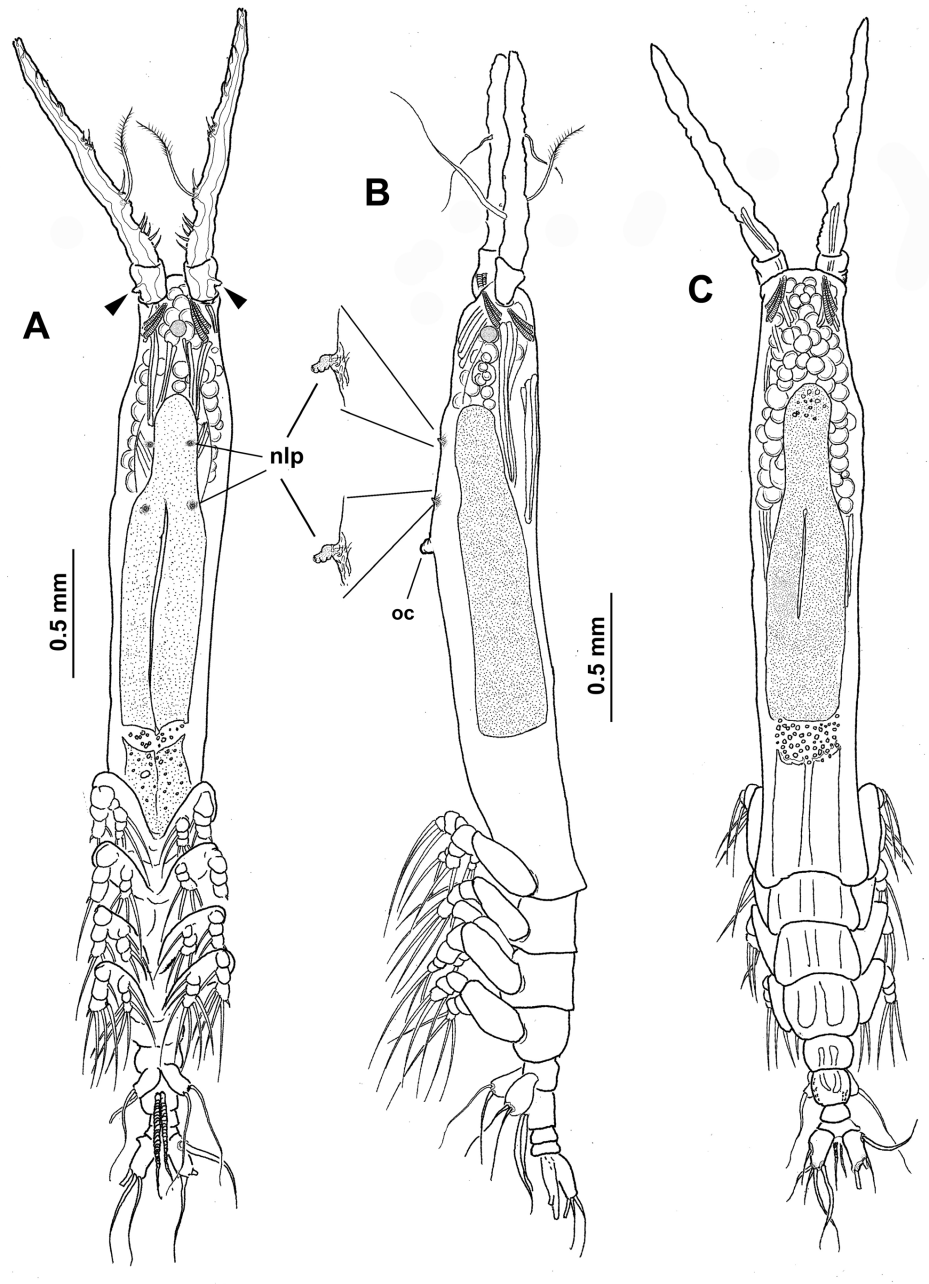
*Monstrilla elongata* Suárez-Morales, 1994 female holotype from the Mexican Caribbean. A. Habitus, ventral; B. Habitus, lateral; C. Habitus, dorsal. Abbreviation: nlp = detail of nipple-like processes.

Body length of holotype 4.2 mm. Cephalothorax robust, cylindrical, representing 58% of total body length, with lateral expansions at distal end of cephalothorax (Fig. 12C; Suárez-Morales 1994: fig. 1A). Oral cone well developed, prominent, papilla-like (Figs 12B, 14D, oc), located 32–35% along ventral surface of cephalothorax from head. Cephalic region of holotype with flat, smooth ‘forehead’ (Fig. 12C); ventral preoral surface with integumental ornamentation consisting of two pairs of nipple-like processes with adjacent integumental wrinkles in both the holotype and paratype (Figs 12A, B, 14A, nlp), field of minute wart-like integumental processes on outer surface of cephalic area adjacent to insertion of antennules (Fig. 15A, B, small arrowheads), and medial preoral pore with adjacent field of integumental wrinkles (Fig. 15B, pop). Eyes comprising two lateral cups (Fig. 15A, lec) and ventral medial cup (Fig. 15A, lec, mec), lateral cups slightly smaller in diameter than medial cup; lateral eye cups weakly pigmented.

**Figure 13.**
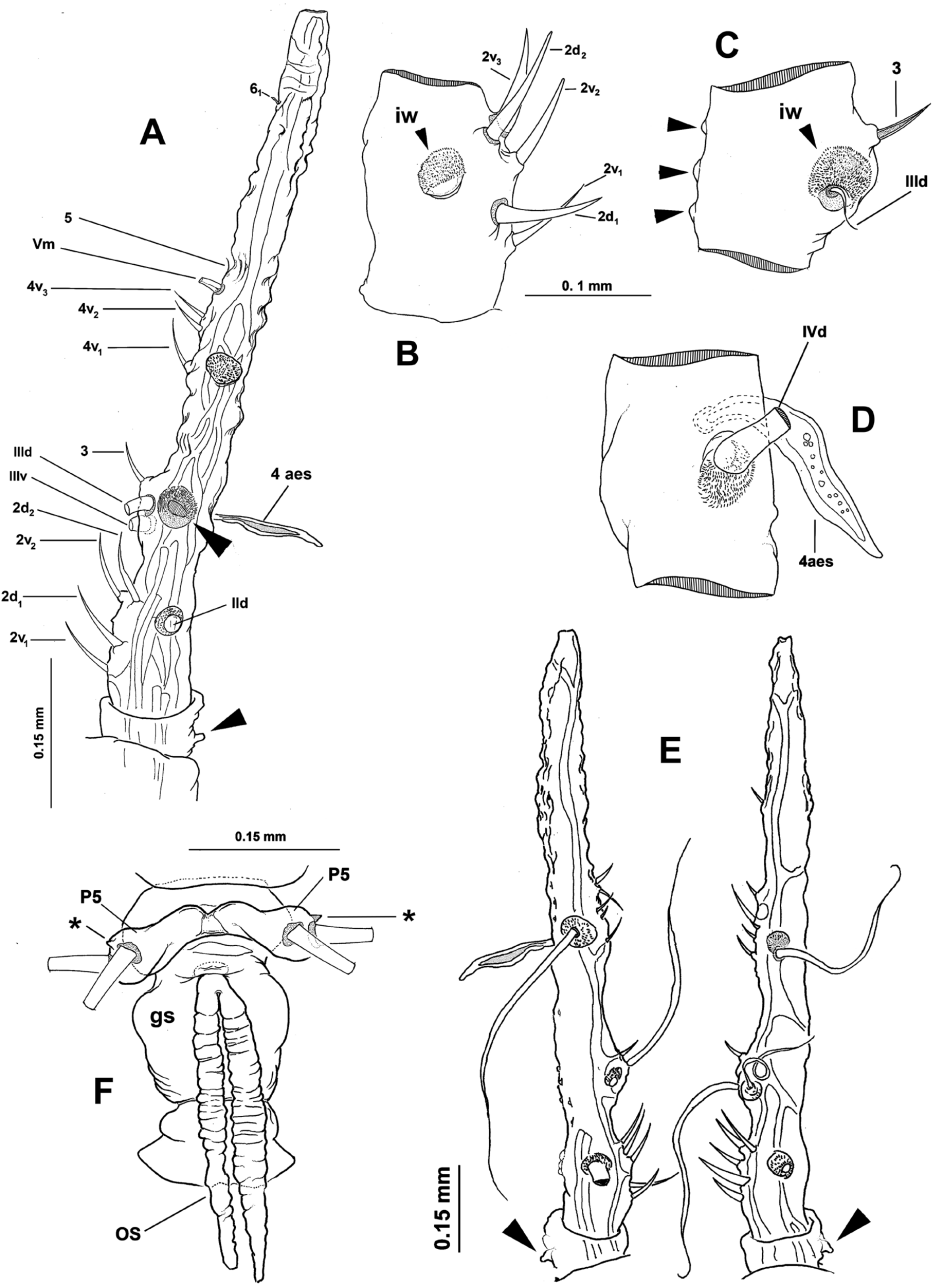
*Monstrilla elongata* Suárez-Morales, 1994 female holotype from the Mexican Caribbean. A. Right antennule, dorsal with setal armature sensu Grygier and Ohtsuka 1995; B. Armature (sensu Grygier and Ohtsuka 1995) of putative second segment of left antennule, dorsal; C. Armature (sensu Grygier and Ohtsuka 1995) of putative third segment of left antennule, dorsal; D. Armature (sensu Grygier and Ohtsuka 1995) of putative fourth antennular segment of left antennule, dorsal; E. Antennules, dorsal (arrowhead indicates lateral thumb-like process) F. Urosome, ventral. Abbreviations: gs = genital double-somite; iw = integumental window; os = ovigerous spine; P5 = fifth legs.

**Figure 14.**
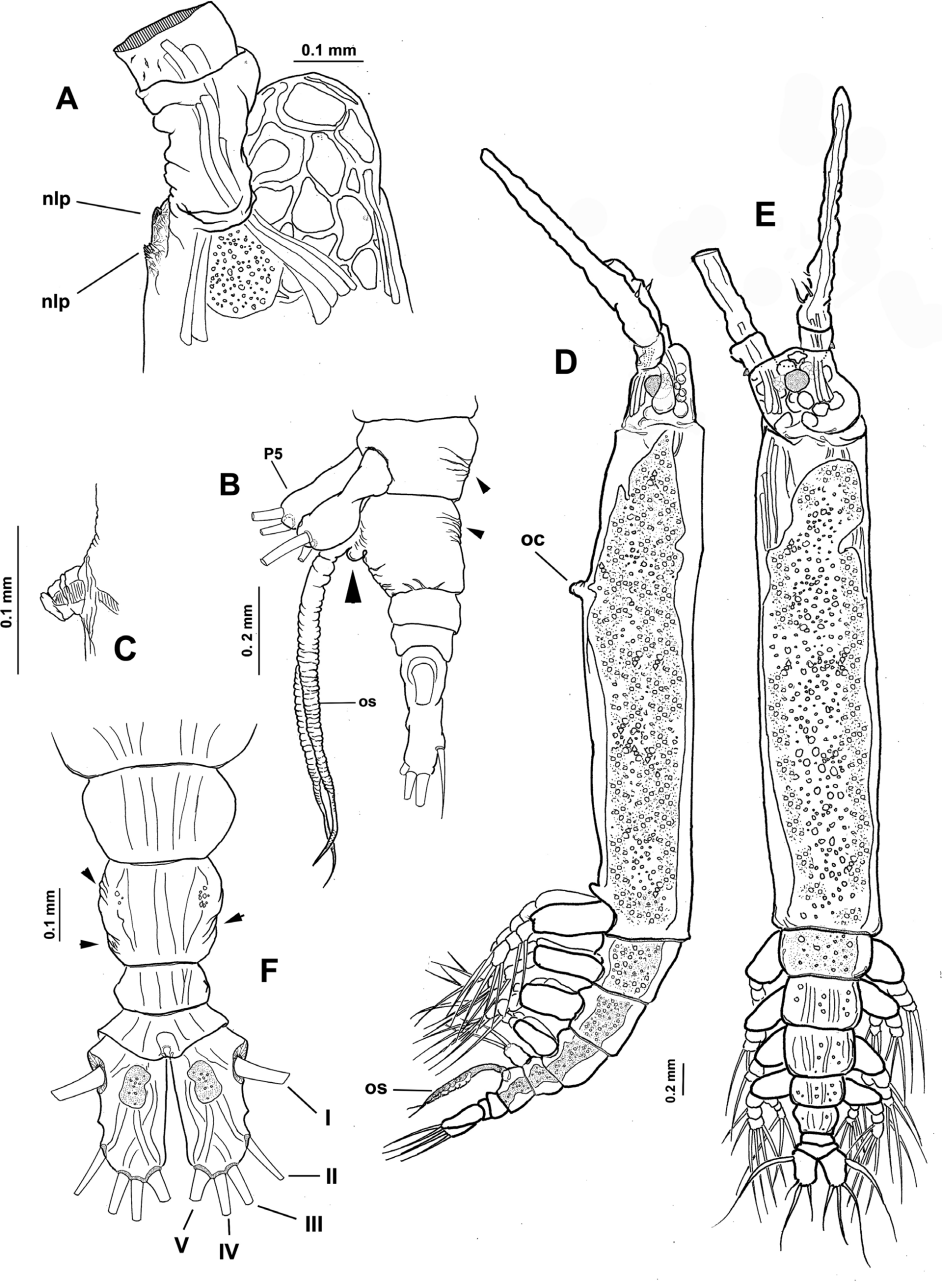
*Monstrilla elongata* Suárez-Morales, 1994 female paratype from the Mexican Caribbean. A. Anterior part of cephalic region, lateral; B. Urosome, lateral (arrowhead indicates ventral genital process); C. Oral cone, detail, lateral; D. Habitus, lateral; E. Habitus, dorsal; F. Urosome, dorsal. Abbreviations: caudal setae I–V sensu Huys and Boxshall 1991; nlp = nipple-like processes; oc = oral cone; os = ovigerous spines; P5 = fifth legs.

**Figure 15.**
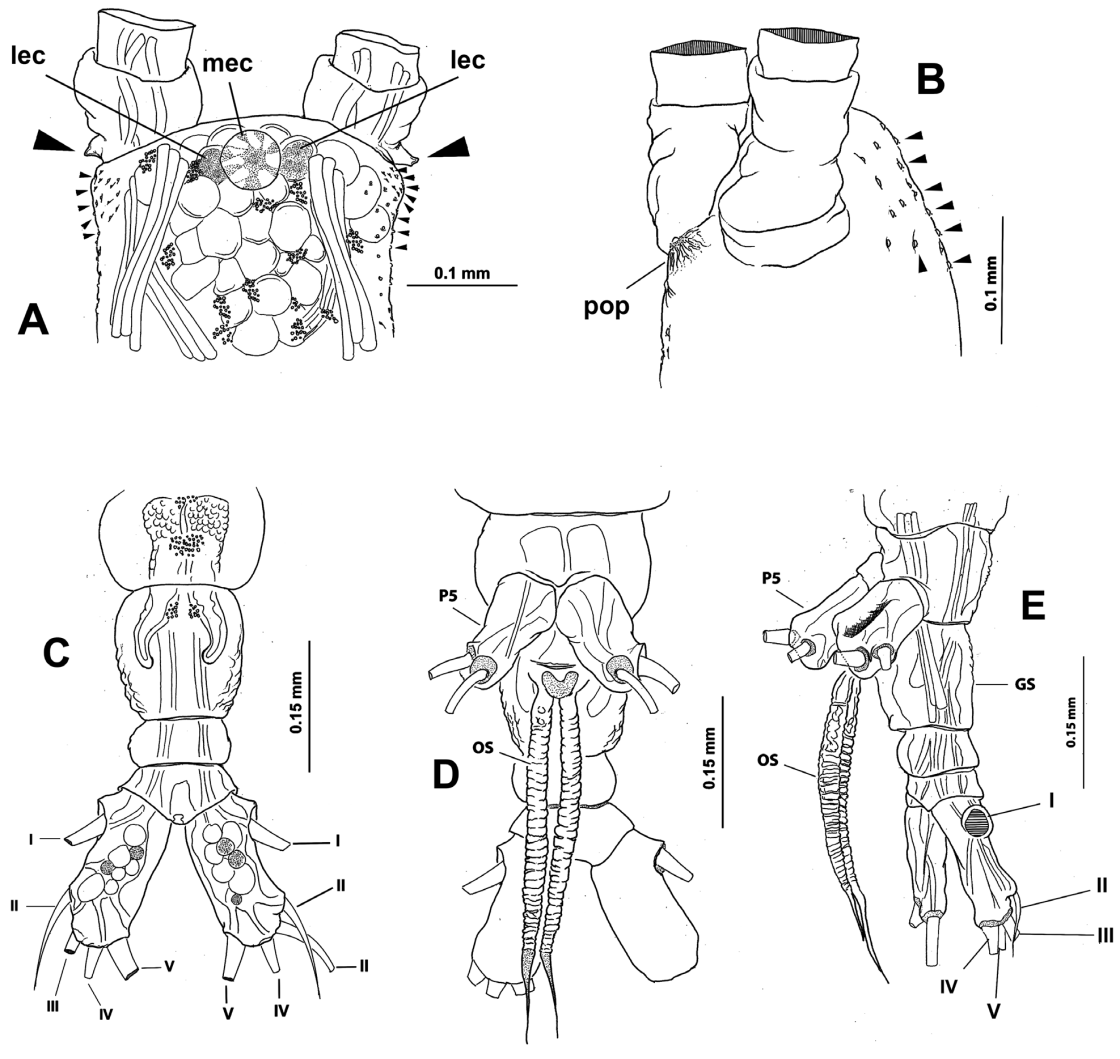
*Monstrilla elongata* Suárez-Morales, 1994 female holotype from the Mexican Caribbean. A. Anterior part of cephalic region (large arrowhead indicates lateral antennular processes; small arrowheads indicate wart-like integumental processes); B. Anterior part of cephalic region; C. Urosome, dorsal; D. Urosome, ventral; E. Urosome, lateral with fifth legs. Abbreviations: caudal setae I–V sensu Huys and Boxshall 1991; lec = lateral eye cup; mec = medial eye cup; os = ovigerous spines; pop = preoral pore complex; P5 = fifth legs.

***Urosome consisting of four somites***: fifth pedigerous somite (carrying fifth legs), genital double-somite with pair of ovigerous spines barely reaching distal margin of caudal rami in holotype (Fig. 12A; Suárez-Morales 1994: fig. 1F), slightly longer in paratype (Fig. 14 B, D), free preanal somite, and anal somite carrying pair of caudal rami; length ratio of urosomites (from proximal to distal) being: 34.91: 37.20: 16.27: 11.62 (Fig. 15D, E). Genital double-somite longest of urosome, with weakly expanded proximal 1/2 ornamented with transverse ridges on dorsal surface; distal 1/2 ornamented with scattered integumental wrinkles in lateral and dorsal surfaces (Figs 14B, F, 15D); pair of relatively short, corrugate ovigerous spines inserted on ventral surface (Figs 13F, 14B, os); spines equally long, both ending in acute, parallel points (Fig. 15D, E). Caudal rami relatively large, subrectangular (Fig. 15C), ~2.5× as long as broad, each ramus armed with five caudal setae (I–V), lateral seta II reduced, being shortest and thinnest (Fig. 15C, E).

Antennules remarkably long, slender, 0.64 mm in length, representing ~23% of total body length and almost 44% of cephalothorax length (Fig. 12A–C); antennules 4-segmented, but segments 2–4 partly fused; antennules weakly divergent in both holotype (Fig. 12A–C) and paratype (Fig. 14D, E) specimens. Intersegmental division between segments 1 and 2 complete (Fig. 13E); length ratio of antennular segments (proximal to distal) 10.71: 25.71: 14.28: 49.3 (Fig. 13E). Following Grygier and Ohtsuka’s (1995) setal nomenclature for female antennules, first segment unarmed, bearing thumb-like process on outer margin (Fig. 13A, E, arrows); putative second segment carrying relatively short spiniform elements 2v_1-3_ and 2d_1,2_, setiform element IId absent, broken off, only sockets observed (Fig. 13B); third segment with slender, stout spiniform element 3 and reduced setiform elements IIId and IIIv (Fig. 13C), fourth segment longest of antennule, proximal 1/2 armed with short spiniform elements 4v_1_, setiform elements IVv and IVd, and aesthetasc 4 aes (Fig. 13D). Distal 1/2 of fourth segment lacking apical armature except for reduced spiniform element 6_1_ in subdistal position (Fig. 13A). Antennules with set of two or three disc-like integumental windows of unknown function in segments 3 and 4 (Fig. 13A–C, iw).

First pedigerous somite and succeeding three free thoracic somites each bearing well-developed pair of biramous swimming legs (Fig. 12A–C), all with exopodite longer than endopodite. Swimming legs 1–4 slender, with setal armature pattern as described by Suárez-Morales 1994: fig. 2J–L). Armature formula of swimming legs 1–4 as:

***Legs Basis Endopod Exopod*** :

Leg 1 1-0 0-1; 0-1; 1, 2, 2 I-1; 0-1; I, 2, 2

Legs 2–4 1-0 0-1; 0-1; 1, 2, 2 I-1; 0-1; I, 2, 3

***Fifth legs*** (Figs 13F, 14B) uniramous, represented by single oblong exopodal segment armed with two equally long terminal setae; legs with subtriangular process adjacent to insertion of setae (Fig. 13F, asterisk). Fifth leg setae long, reaching distal margin of caudal rami (Fig. 12A–C; Suárez-Morales 1994: figs 1F, 2H).

**Male.** Unknown.

##### Remarks.

In the original description of this species, Suárez-Morales (1994) did not provide information about the eye cups morphology, which are indeed not easily discernible in the type specimens. Following Isaac’s (1975) criteria, the author incorrectly mentioned the absence of eyes as a character of the genus *Monstrilla*. The eye structure is described herein based on the re-examination of the type specimens.

According to Suárez-Morales (1994), the main character to distinguish this species from its congeners is the fifth leg structure and armature, consisting of a single segment armed with two setae, a character shared with several congeners like *M. conjunctiva* Giesbrecht, 1902, *Caromiobenella helgolandica* Claus, 1863 (= *M. helgolandica*), *M. longipes* Scott, 1909 (assigned to *Maemonstrilla* by Grygier and Ohtsuka 2008), and *M. ghardaqensis* Al-Kholy, 1963, likely a synonym of *C. helgolandica* (pers. obs.). Additional species of *Monstrilla* with the same fifth leg armature pattern include the recently described *M. annulata* Suárez-Morales, 2024, from a Mexican Caribbean reef system (see Suárez-Morales 2024), *M. leucopis* Sars, 1921 from Norway and the Gulf of California (Suárez-Morales 2010; Suárez-Morales and Velázquez-Ornelas 2023), and *M. wandelii* from Greenland (Park 1967). The antennule length is a helpful character to separate species of this group. In *M. leucopis*, *M. annulata*, and *M. elongata* antennules are long and slender, representing almost 50% (*M. elongata*) or even >60% (i.e., *M. leucopis*, *M. annulata*) of the cephalothorax length (Suárez-Morales and Velázquez-Ornelas 2023; Suárez-Morales 2024). Among these three species, only *M. elongata* exhibits a distinctive set of disc-like integumental processes (Fig. 13A–C). Furthermore, the recently described *M. mahahualensis* Suárez-Morales, 2022, based on male specimens, shares with *M. elongata* two interesting characters of the antennules: the presence of disc-like integumental processes and the absence of antennular element 1 (sensu Grygier and Ohtsuka 1995). In males of *M. mahahualensis* the disc-like integumental processes are present in antennular segments 3 and 4 (Suárez-Morales 2022: fig. 1C, E, F), whereas in *M. elongata*, these processes are also found on the second segment (Fig. 13B). The absence of antennular element 1 has been also reported in other species of *Monstrilla* like *M. wandelii*, *M. leucopis*, *M. annulata*, *M. conjunctiva*, and *M. parki* (see Suárez-Morales 2024; Suárez-Morales and McKinnon 2025), but only in *M. elongata* the first segment has a thumb-like outer process (Fig. 13A, E, arrows). Also, *M. annulata* has a distinctively annulated antennule and an hirsute fifth leg (Suárez-Morales 2024), thus diverging from *M. elongata*.

#### ﻿ Monstrilla gibbosa

Taxon classificationAnimaliaMonstrilloidaMonstrillidae

Suarez-Morales & Palomares-Garcia, 1995

CA69C0CF-C533-58CD-82B4-87C35B32D2FF

**Figure 16.**
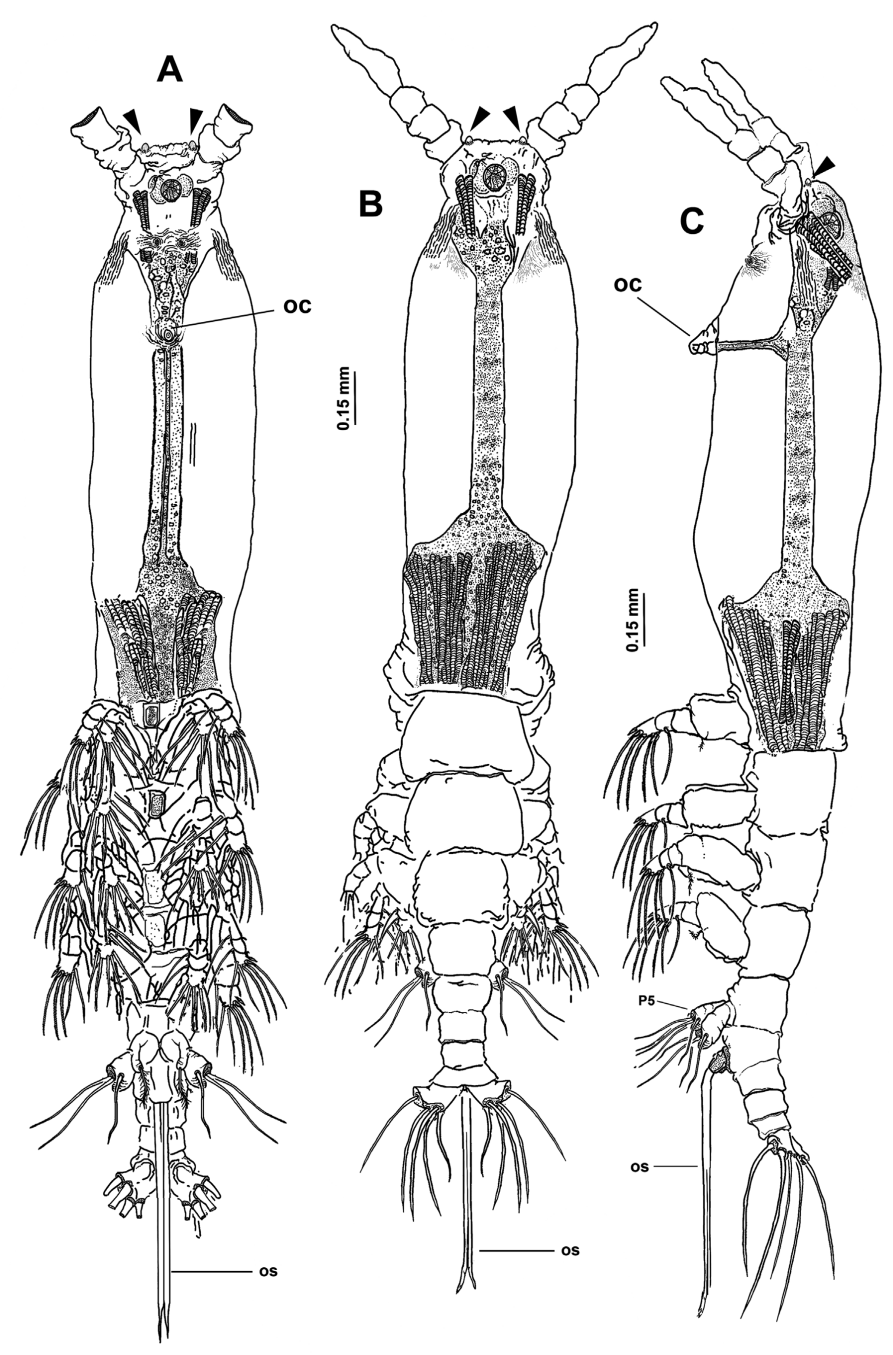
*Monstrilla gibbosa* Suárez-Morales & Palomares-Garcia, 1995 female holotype from the Gulf of California. A. Habitus, ventral; B. Habitus, dorsal; C. Habitus, lateral. Abbreviation: oc = oral cone.

**Figure 17.**
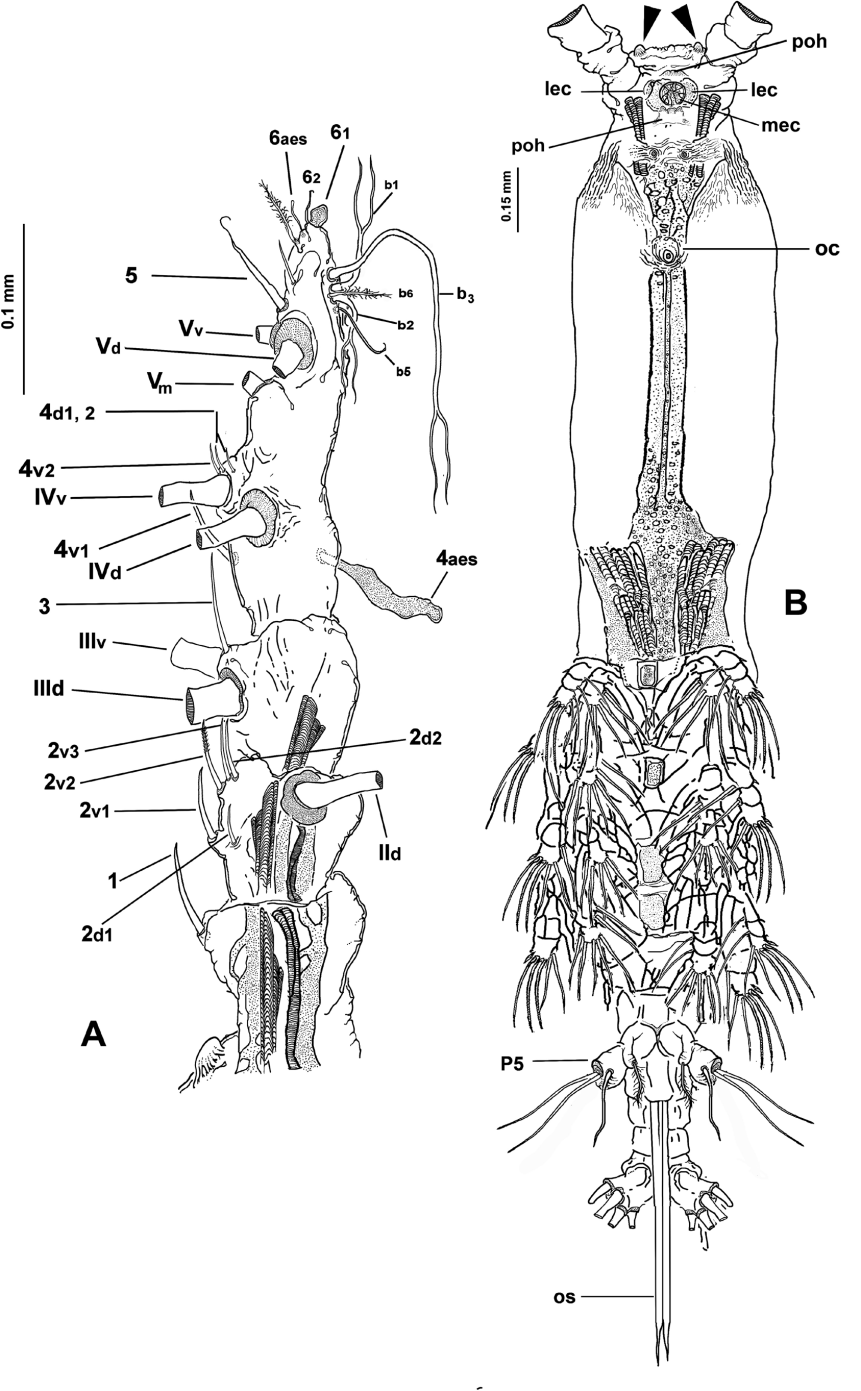
*Monstrilla gibbosa* Suárez-Morales & Palomares-Garcia, 1995 female holotype from the Gulf of California. A. Right antennule, dorsal with armature (sensu Grygier and Ohtsuka 1995); B. Habitus, ventral. Abbreviation: oc = oral cone.

**Figure 18.**
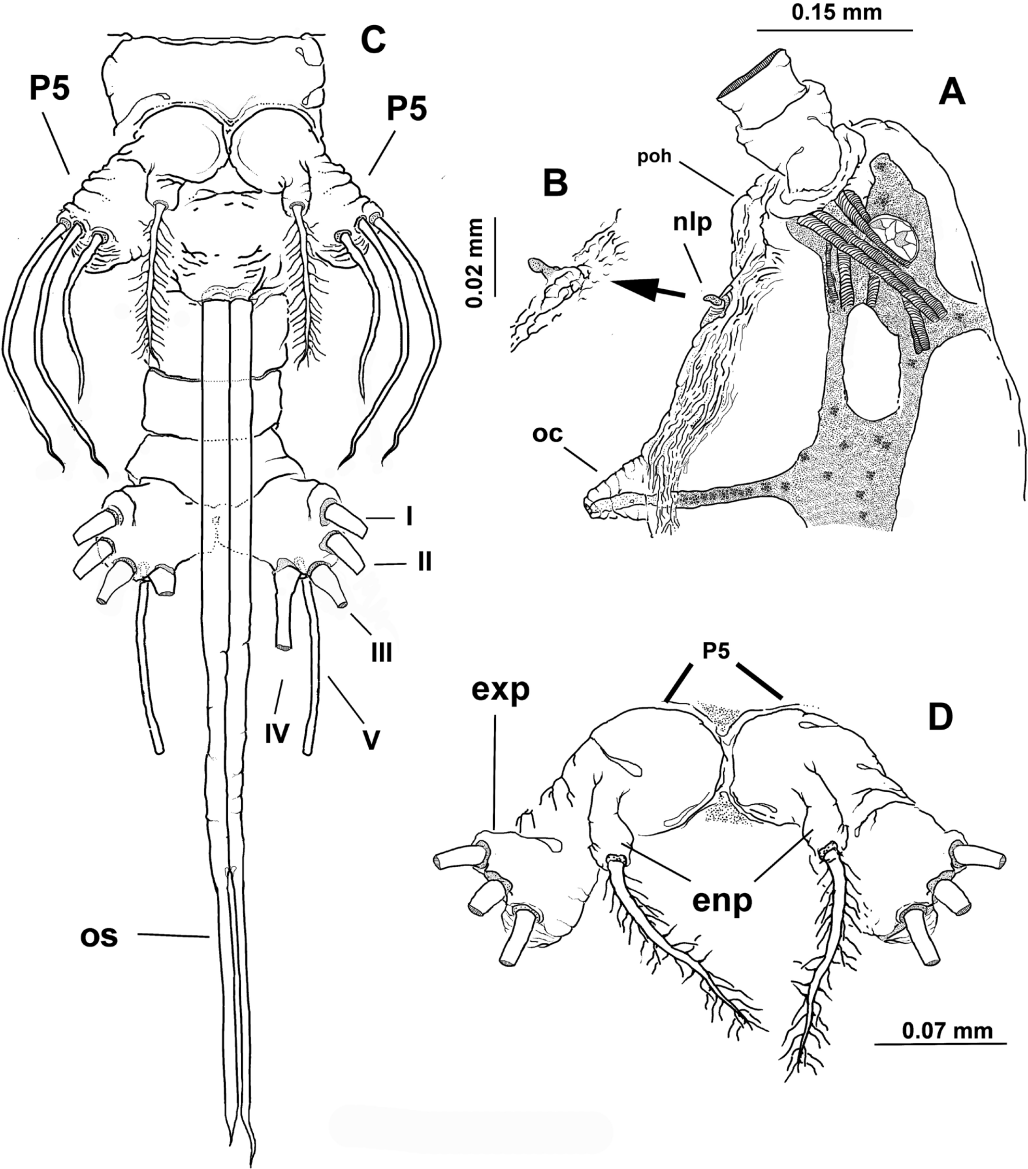
*Monstrilla gibbosa* Suárez-Morales & Palomares-Garcia, 1995 female holotype from the Gulf of California. A. Cephalic region, lateral; B. Detail of nipple-like process; C. Urosome, ventral; D. Fifth legs, ventral. Abbreviations: caudal setae I–V sensu Huys and Boxshall 1991; enp = endopod; exp = exopod; nlp = nipple-like process; P5 = fifth legs.

**Figure 19.**
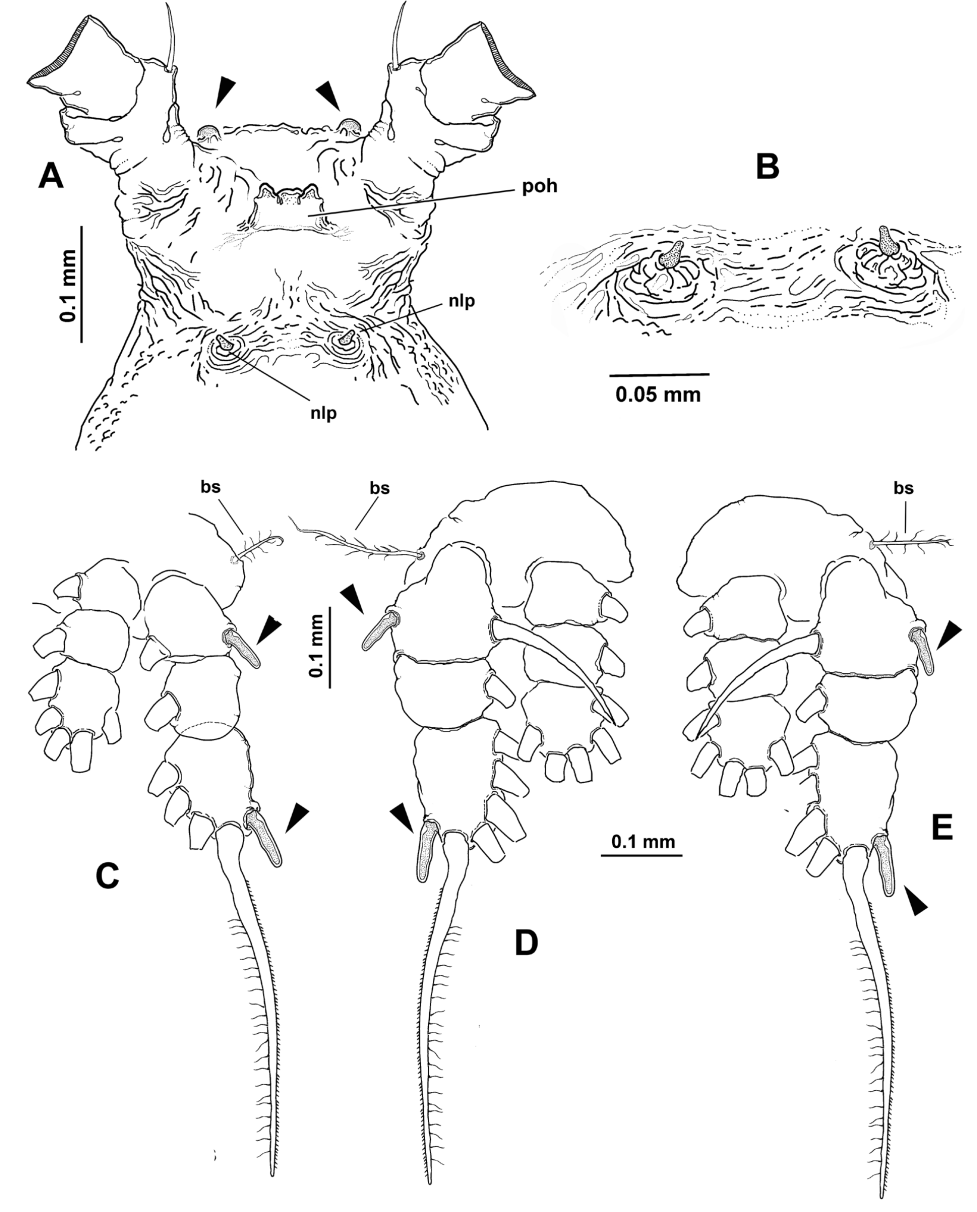
*Monstrilla gibbosa* Suárez-Morales & Palomares-Garcia, 1995 female holotype from the Gulf of California. A. Anterior part of cephalic region, ventral (arrowheads indicate frontal rounded processes); B. Detail of nipple-like processes, ventral; C. First swimming leg, anterior; D. Third swimming leg, anterior; E. Second swimming leg, anterior. Abbreviations: bs = basipodal seta; nlp = nipple-like processes.

**Figure 20.**
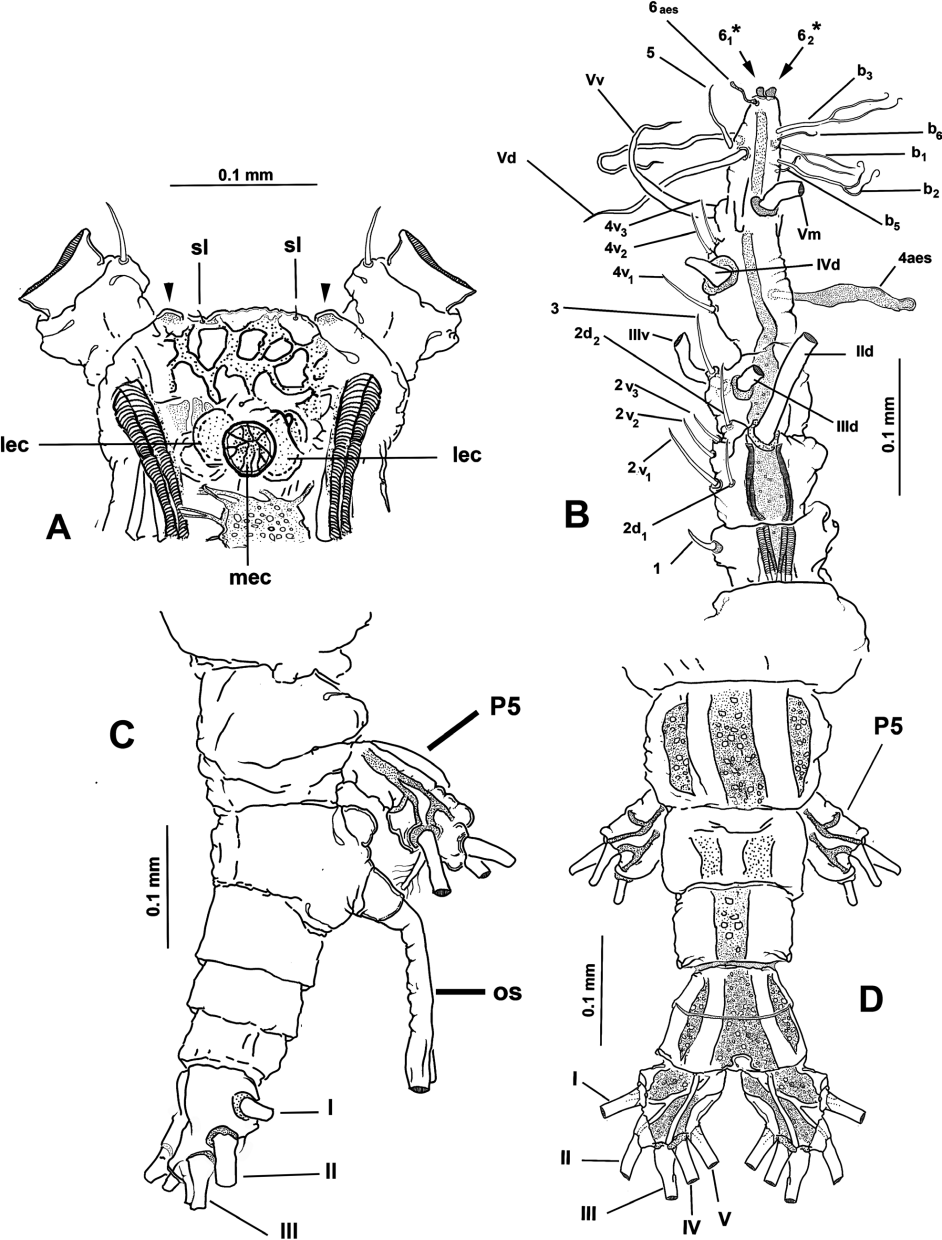
*Monstrilla gibbosa* Suárez-Morales & Palomares-Garcia, 1995 female holotype from the Gulf of California. A. Anterior part of cephalic region, dorsal; B. Right antennule, dorsal with setal armature sensu Grygier and Ohtsuka 1995; C. Urosome, lateral; D. Urosome, dorsal. Abbreviations: caudal setae I–V sensu Huys and Boxshall 1991; lec = lateral eye cup; mec = medial eye cup; os = ovigerous spines; P5 = fifth legs; sl = sensilla.

Figs 16, 17, 18, 19, 20

##### Type material.

***Holotype*** • adult female, undissected, deposited in the collection of Crustacea, U.S, National Museum of Natural History, Smithsonian Institution, USNM 259488. Adult female paratype USNM-259665.

##### Type locality.

Puerto Escondido coastal system, southern part of the eastern coast of the Baja California Peninsula, Mexico (25°49'N, 111°18'W). Date of collection 18 November 1993.

##### Description of adult female holotype.

Body length of holotype 4.2 mm. Cephalothorax moderately robust, representing almost 60% of total body length, with lateral expansions at distal end of cephalothorax (Fig. 16A–C; Suárez-Morales and Palomares-García 1995: fig. 1a). Oral cone well developed, prominent, papilla-like (Figs 17B, 18A, oc), located at 32% on ventral surface of cephalothorax. Cephalic region with weakly produced, lightly rugose ‘forehead’ (Figs 16B, 17B) with pair of small spherical processes near insertion of antennules (Figs 16A–C, 17B, 19A, arrowheads); forehead with pair of minute sensilla (Fig. 20A, sl). Ventral preoral surface with medially divided hump-like medial protuberance (Figs 17B, 18A, 19A, poh). Integumental ornamentation consisting of single pair of well-developed nipple-like processes with adjacent integumental wrinkles (Figs 18A, B, 19B, nlp), and field of minute longitudinal wrinkles stretching ventrally between nlps and oral cone (Figs 17B, 18A). Eyes comprising two lateral cups (Figs 17B, 20A, lec) and ventral medial cup (Figs 17B, 20A, mec), lateral and medial cups with approximately the same diameter, eyes weakly pigmented.

***Urosome consisting of four somites***: fifth pedigerous somite (carrying fifth legs), genital double-somite with pair of ventral ovigerous spines reaching well beyond distal margin of caudal rami (Fig. 18C; Suárez-Morales and Palomares-García 1995: fig. 1B), free preanal somite, and anal somite carrying pair of caudal rami; length ratio of urosomites (from proximal to distal) being: 36.66: 39.96: 13.98: 9.49 (Figs 1C, 20C). Genital double-somite longest of urosome, with weakly expanded proximal 1/2 ornamented with transverse ridges on dorsal surface; distal 1/2 ornamented with scattered integumental wrinkles in lateral and dorsal surfaces (Figs 18C, 20C); pair of long, slender ovigerous spines inserted on ventral surface (Figs 16A–C, 17B, 18C, os); spines basally conjoined, equally long, both ending in acute, parallel points. Caudal rami subquadrate (Fig. 18C), ~1.3× as long as broad, each ramus armed with five caudal setae (I–V), proximal outer seta I longest, outer terminal seta III proximally expanded, seta V shortest, slender, inserted dorsally (Figs 18C, 20C, D).

Antennules relatively robust, slender, divergent, 0.42 mm in length, ~20% of total body length and almost 35% of cephalothorax length (Fig. 16B, C); antennules 4-segmented, segments 3 and 4 partly fused; Intersegmental division between segments 1 and 2 and 2 and 3 complete (Fig. 17A); length ratio of antennular segments (proximal to distal) 19.20: 16.55: 17.88: 46.36 (Fig. 20B). Following Grygier and Ohtsuka’s (1995) setal nomenclature for female antennules, first segment armed with slender, spiniform element 1; second segment carrying complete armature comprising slender spiniform elements 2v_1-3_ and 2d_1,2_, and setiform element IId (Figs 17A, 20B); third segment with slender, stout spiniform element 3 and setiform elements IIId and IIIv (Fig. 17A), fourth segment longest of antennule, proximal 1/2 armed with short, slender spiniform elements 4v_1,2_, setiform elements IVv and IVd, and aesthetasc 4 aes (Figs 17A, 20B). Distal 1/2 of fourth segment armature comprising setiform elements Vd, Vm, Vd, and spiniform element 5; apical armature including apical aesthetasc 6aes, apical spine 6_1, 2_, the former being short and blunt. Outer distal margin with setae of the “b-group”; b_1-3,5,6_, only b_1_, b_3_ branched (Fig. 20B).

First pedigerous somite and succeeding three free thoracic somites each bearing well-developed pair of biramous swimming legs (Fig. 19C–E), all with exopod longer than endopod. Swimming legs 1–4 robust, with setal armature pattern as described by Suárez-Morales and Palomares-García (1995) (Fig. 19C–E). Exopodal spines of legs 1–4 apically rounded (Fig. 19C–E, arrowheads). Basipodal seta of third swimming leg longest (Fig. 19D) Armature formula of swimming legs 1–4 as:

***Legs Basis Endopod Exopod*** :

Leg 1 1-0 0-1; 0-1; 1, 2-2 I-1; 0-1; I, 2, 2

Legs 2–4 1-0 0-1; 0-1; 1, 2, 2 I-1; 0-1;I, 2, 3

***Fifth legs*** (Figs 18C, D, 20C, D) biramous, represented by large, weakly corrugate subrectangular exopodal segment armed with three equally long terminal setae (Fig. 18C, D). Endopod represented by small thumb-like lobe armed with short distal seta (Fig. 18D; Suárez-Morales and Palomares-García 1995: fig. 2a). Fifth leg setae long, reaching proximal margin of caudal rami (Fig. 18C; Suárez-Morales and Palomares-García 1995: figs 2a, 3a, b).

**Male.** Unknown.

##### Remarks.

In the original description of this species, Suárez-Morales and Palomares-García (1995) emphasized the structure and armature of the fifth leg as the main distinctive character of *M. gibbosa*. They compared it with several other congeneric species sharing a 3 exopodal-1 endopodal fifth leg setal armature, like *M. turgida* Scott, 1909, *M. reticulata* Davis, 1949, *M. longicornis* Thompson, 1890, *M. lata* Desai & Bal, 1963, and *M. barbata*. Details of the fifth leg lobes and setae were compared among species of this group to reliably recognize *M. gibbosa*. In addition, Suárez-Morales and McKinnon (2025) included *M. gibbosa* among the species of *Monstrilla* with a relatively long fifth leg endopodal lobe, together with the Baja Californian *M. hendrickxi* Suárez-Morales & Velázquez-Ornelas, 2024 and the Australian *M. janetgrieveae* Suárez-Morales & McKinnon, 2025; the inner lobe of *M. gibbosa* is clearly the shortest among these species, barely reaching the midlength of the exopodal lobe inner margin; in addition, the endopodal lobe is unarmed in *M. hendrickxi*. The antennules length and segmental fusion patterns were also evaluated to distinguish *M. gibbosa*. It was also noticed that the rhomboid shape of the apical spiniform antennular element 6_1_ (*sensu*Grygier and Ohtsuka 1995) is unique among the compared species of *Monstrilla*. The presence of an antero-ventral hump-like cephalic process was described as the main distinctive character of *M. gibbosa*; in fact, this process, previously described as two processes, is a single bipartite one (Fig. 19A), but not discernible as such in lateral view (Fig. 18A). Another unique character of this species is the presence of a pair of globular processes in the frontal area adjacent to the antennules insertion; this kind of process has not been described in other known species of *Monstrilla*. It was depicted by Suárez-Morales and Palomares-García (1995: fig. 3d) but was not included in the description. Also, Suárez-Morales and Palomares-García (1995: fig. 2b, c) depicted the antennular setal armature as comprising three branched setae, but there are only two branched setae (b_1_, b_3_) in the holotype (Figs 17A, 20B). A set of branched “b-group” setae has been reported also in other species of the genus like *M. grandis* Giesbrecht, 1891, *M. bahiana* Suárez-Morales & Dias, 2001, *M. bernardensis* Davis & Green, 1974 (Suárez-Morales and McKinnon 2025) and recently also in the Chinese *M. pseudograndis* Zhou, Lian & Tan, 2025.

In Suárez-Morales and Palomares-García (1995: fig. 2a), the fifth leg exopodal setae are depicted as having a relatively shorter middle exopodal seta, whereas the innermost seta is in fact the shortest in the holotype (Figs 17B, 18C). Another relevant character that was depicted (Suárez-Morales and Palomares-García (1995: figs 1a, 3b) but not described is the presence of a proximally swollen caudal seta (seta III) in *M. gibbosa* (Figs 18C, 20D). According to Suárez-Morales and McKinnon (2025), several Australian species of *Monstrilla* exhibit proximally swollen caudal setae (setae III and IV), thus diverging from *M. gibbosa*, exhibiting a single swollen seta.

#### ﻿ Monstrilla mariaeugeniae

Taxon classificationAnimaliaMonstrilloidaMonstrillidae

Suárez-Morales & Islas-Landeros, 1993

8ED07EE7-A5FB-5F59-90D6-7295310A67C4

Figs 21, 22, 23

##### Material examined.

***Holotype***

##### •

female, vial deposited at the USNM-Smithsonian Institution under number USNM-251840. Paratypes: adult female, vial deposited at the USNM (USNM-251841), Washington D.C. Additional paratypes, seven adult females, deposited in the Collection of Zooplankton at El Colegio de la Frontera Sur (ECOSUR) in Chetumal, Mexico. Types preserved in ethanol.

##### Type locality.

Reef lagoon off Puerto Morelos, state of Quintana Roo, northern part of the Yucatan Peninsula eastern coast (20°15.5'N, 86°54.5'W). Water column. Depth 1.2 m.

##### Description of adult female holotype.

**Figure 21.**
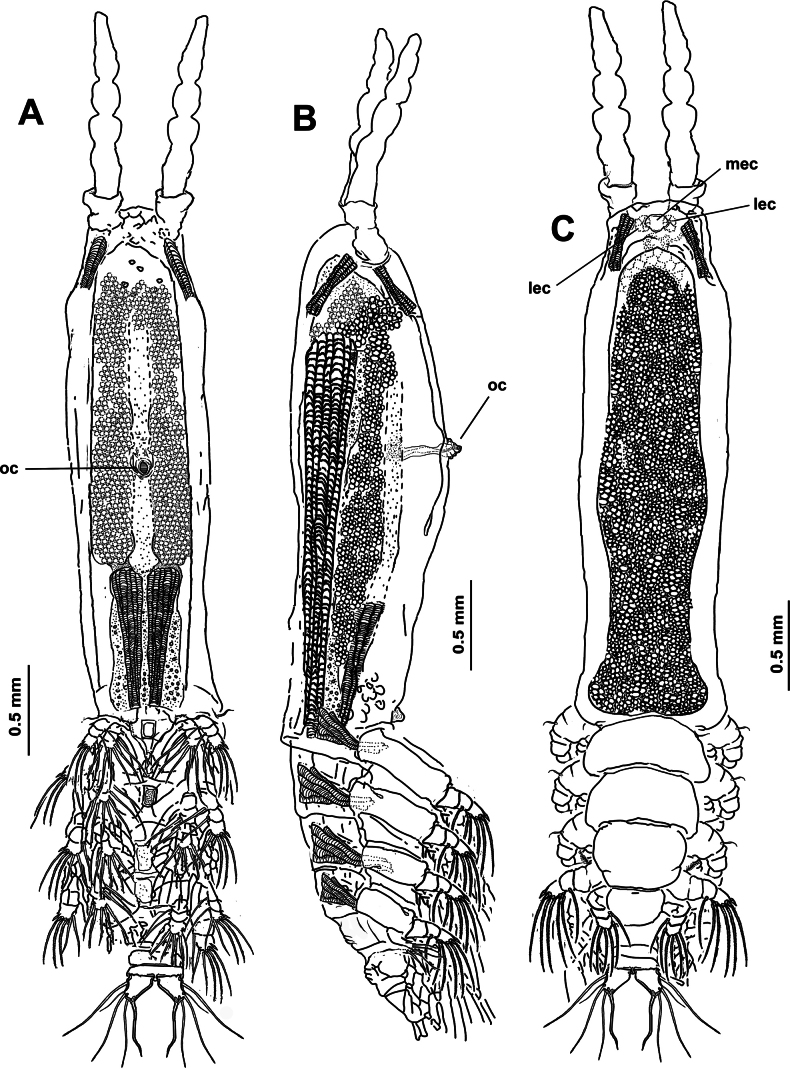
*Monstrilla mariaeugeniae* Suárez-Morales & Islas-Landeros, 1993, female holotype from the Mexican Caribbean. A. Habitus, ventral; B. Habitus lateral; C. Habitus, dorsal.

**Figure 22.**
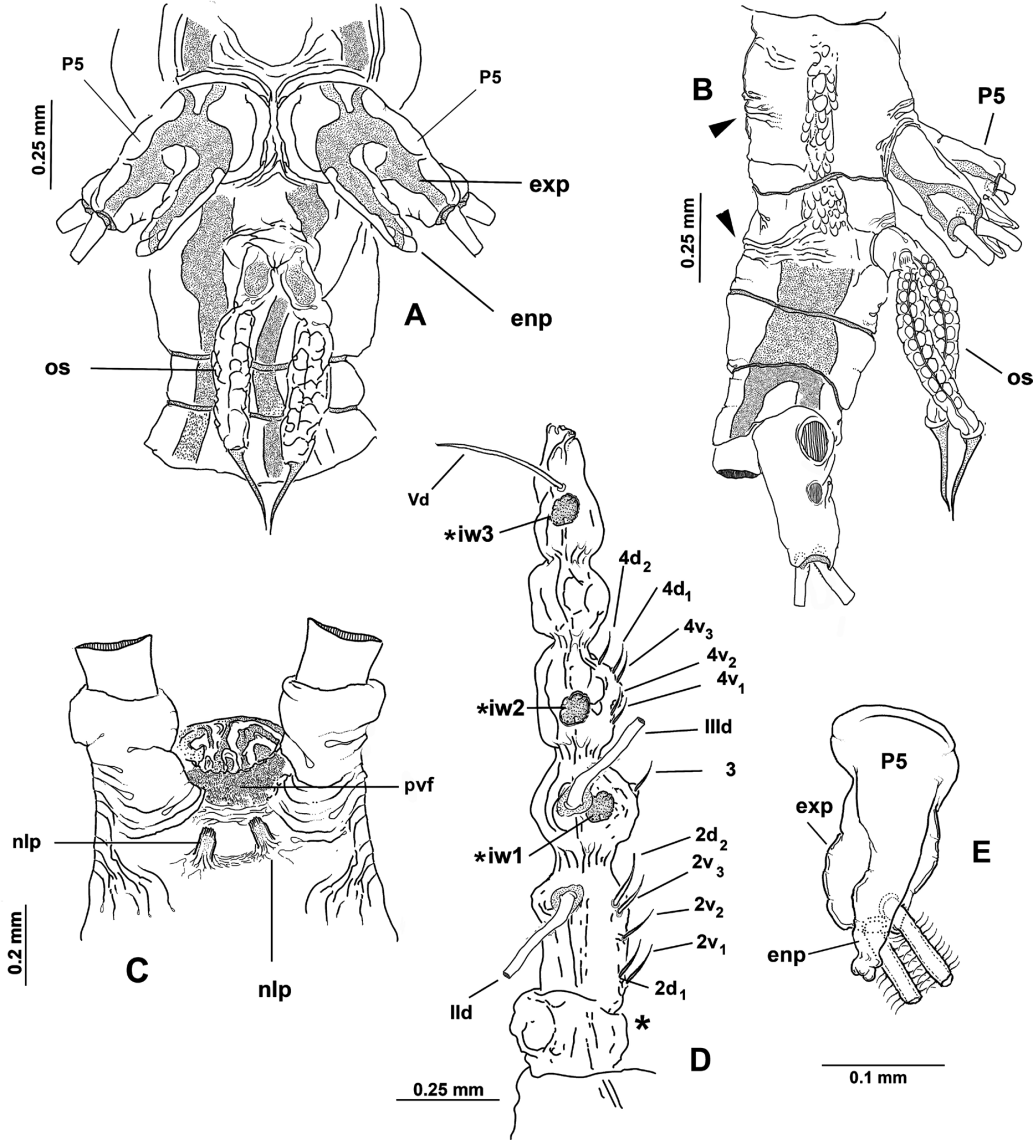
*Monstrilla mariaeugeniae* Suárez-Morales & Islas-Landeros, 1993, female holotype from the Mexican Caribbean. A. Urosome, ventral B. Urosome, lateral (arrowheads indicate dorsal integumental wrinkles); C. Anterior part of cephalic region, ventral; D. Left antennule with setal armature (sensu Grygier and Ohtsuka 1995); E. Fifth leg, lateral. Abbreviations: en = endopod; exp = exopod; nlp = nipple-like processes; os = ovigerous spines; pvf = pilose ventral field.

Body length of holotype 4.5 mm, paratypes body length ranging between 4.2 and 4.7 mm. Cephalothorax long, robust, representing almost 66% of total body length, with lateral expansions at distal end of cephalothorax (Fig. 21A–C; Suárez-Morales and Islas-Landeros 1993: fig. 1a). Oral cone weakly developed, papilla-like (Fig. 21A, B, oc), located at 43% along ventral surface of cephalothorax. Cephalic region with weakly produced, lightly rugose ‘forehead’ (Fig. 22C) with pilose integumental field between antennules insertion (Fig. 22C, pvf); ventral preoral surface with single pair of protuberant nipple-like processes with adjacent integumental wrinkles (Fig. 22C, nlp; Suárez-Morales and Islas-Landeros, 1993: fig. 1k), and adjacent field of minute integumental wrinkles. Eyes weakly pigmented, comprising two lateral cups (Fig. 21C, lec) and ventral medial cup (Fig. 21C, mec), medial cup slightly larger than lateral cups.

**Figure 23.**
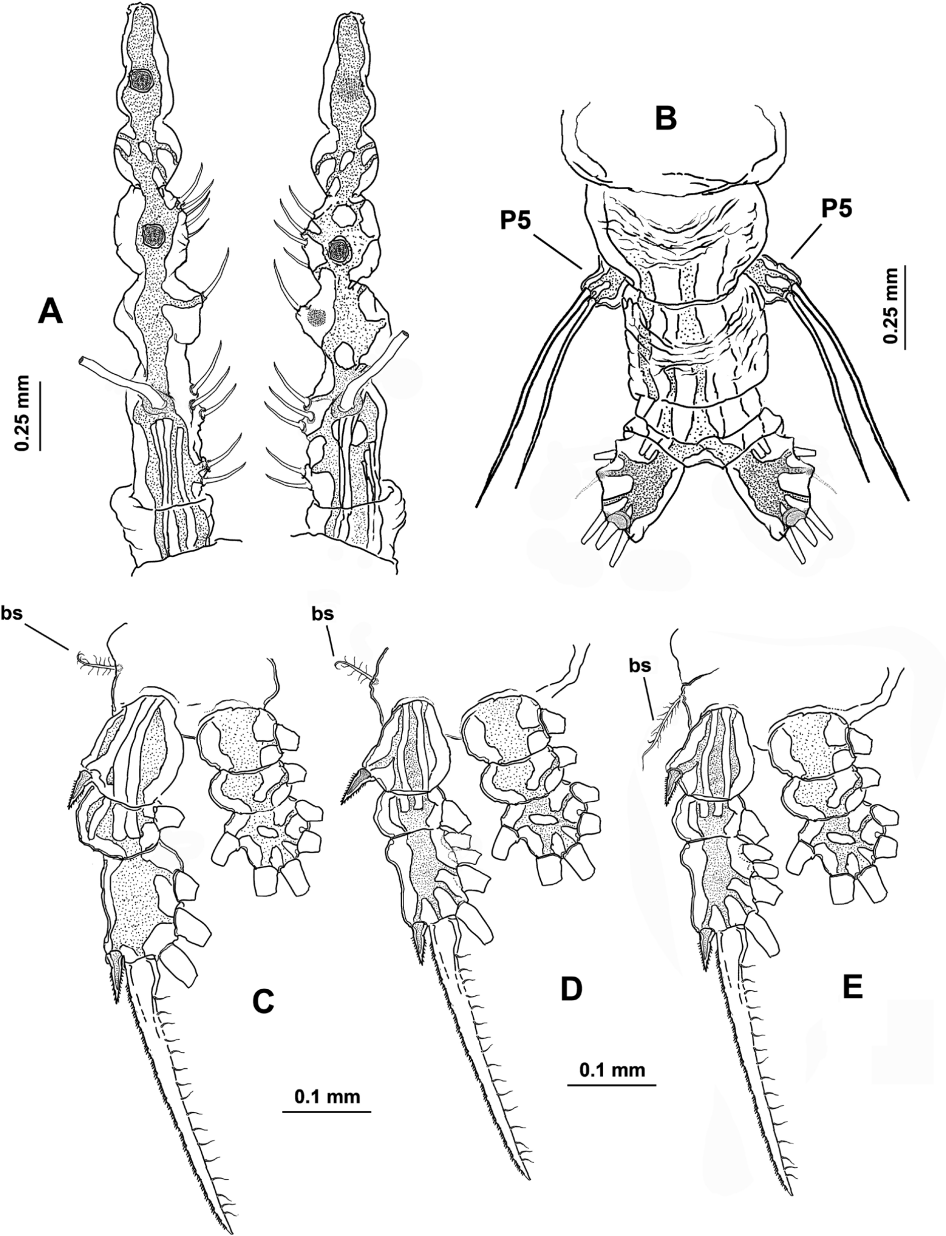
*Monstrilla mariaeugeniae* Suárez-Morales & Islas-Landeros, 1993, female holotype from the Mexican Caribbean. A. Antennules, dorsal; B. Urosome, dorsal; C. First swimming leg, anterior; D. Second swimming leg, anterior; E. Third swimming leg, anterior. Abbreviations: bs = basipodal seta; caudal setae I–IV sensu Huys and Boxshall 1991; P5 = fifth leg.

***Urosome consisting of four somites***: fifth pedigerous somite (carrying fifth legs), genital double-somite with pair of ovigerous spines (Fig. 22A, B; Suárez-Morales and Islas-Landeros, 1993: fig. 1e, f), free preanal somite, and anal somite carrying pair of caudal rami; length ratio of urosomites (from proximal to distal) being: 40.24: 24.39: 20.73: 14.64 (Fig. 22A, B). Genital double-somite with weakly expanded lateral margins, dorsal surface ornamented with transverse integumental ridges (Figs 22B, 23B, arrowhead). Ovigerous spines short, sausage-like, inserted on ventral surface (Fig. 22B, os), barely reaching distal margin of anal somite (Fig. 22A, B); spines distally tapering into acute, parallel points. Caudal rami subquadrate (Fig. 23B), ~1.2× as long as broad, each ramus armed with five caudal setae (I–V), proximal outer seta I longest, outer seta II reduced, shortest (Fig. 23B).

Antennules relatively robust, slender, parallel, ~28% of total body length and almost 43% of cephalothorax length (Fig. 21A, C); antennules 4-segmented, segments 2–4 partly fused; proximalmost intersegmental division complete (Figs 22D, 23A); divisions between successive segments marked by strong constrictions (Fig. 22D). Length ratio of antennular segments (proximal to distal, identified by segmental armature sensu Grygier and Ohtsuka 1995): 11.21: 23.36: 15.88: 49.55 (Figs 22D, 23A). Following Grygier and Ohtsuka’s (1995) setal nomenclature for female antennules, first segment unarmed, element 1 absent (Fig. 22D, asterisk); second segment carrying complete armature comprising short, slender spiniform elements 2v_1-3_ and 2d_1,2_, and setiform element IId (Figs 22D, 23A); third segment with slender, curved spiniform element 3 and setiform element IIId; element IIIv not observed (Fig. 22D); third segment with disk-shaped integumental window (Fig. 22D, iw1). Fourth segment longest of antennule, showing two additional constrictions. Proximal 1/3 of segment armed with short, slender spiniform elements 4v_1,2_, 4d_1,2_ and ornamented with disk-like integumental window (Fig. 22D, iw2). Armature of fourth segment distal 1/2 reduced, comprising setiform element Vd only; third disk-like integumental window present on this segment (Fig. 22D, iw3). Apical armature not observable in type specimens, possibly broken off during collection.

First pedigerous somite and succeeding three free thoracic somites each bearing well-developed pair of biramous swimming legs (Fig. 23C–E), all with exopod longer than endopod. Legs 2–4: identical in size and setation, carrying outer basipodal seta (Fig. 23C–E, bs); basipodal seta longest in leg 3 (Fig. 23E). Swimming legs 1–4 with relatively short, robust apical spiniform setae on the third exopod. Setal armature pattern as described by Suárez-Morales and Islas-Landeros (1993) (see also Fig. 23C–E). Armature formula of swimming legs 1–4 as:

***Legs Basis Endopod Exopod*** :

Leg 1 1-0 0-1; 0-1; 1, 2, 2 I-1; 0-1; I, 2, 2

Legs 2–4 1-0 0-1; 0-1; 1, 2, 2 I-1; 0-1; I, 2, 3

***Fifth legs*** (Figs 22A, B, E, 23B) biramous, represented by large, smooth subrectangular exopodal segment armed with two equally long terminal setae (Figs 22A, 23B). Endopod represented by long digitiform, unarmed inner lobe reaching distal margin of exopodal lobe (Fig. 22A). Fifth leg setae long, barely reaching distal margin of caudal rami (Fig. 23B; Suárez-Morales and Islas-Landeros 1993: fig. 1a, e, h).

**Male.** Unknown (see further comments in Suárez-Morales 2022).

##### Remarks.

*Monstrilla mariaeugeniae* is the largest (body length = 4.2–4.7 mm) monstrilloid copepod reported from the Mexican Caribbean, followed by *M. elongata* (4.2 mm) and *M. xcalakensis* (3.51 mm). In the original description this species was considered to be closely related with *M. wandelii*. The first character used to separate both species was the lack of basipodal setae on swimming legs 1–4 in *M. mariaeugeniae* (Suárez-Morales and Islas-Landeros 1993); this is erroneous because this species has basipodal setae in all swimming legs, as revealed in this redescription (Fig. 23C–E). The antennular constrictions exhibited by *M. mariaeugeniae* were also mentioned as a relevant character to separate *M. wandelii* and *M. mariaeugeniae* (Suárez-Morales and Islas-Landeros 1993). Also, the size of *M. mariaeugeniae* females (4.4–4.7 mm) is nearly twice of that known for *M. wandelii*.

In their revision of the Australian species of *Monstrilla*, Suárez-Morales and McKinnon (2025) realized that there is a group of species related to *M. conjunctiva* Giesbrecht, 1902 comprising several species: (1) *M. wandelii*, (2) male specimens designated by Park (1967) as *M. wandelii* (but see Suárez-Morales 2022 for further comments), (3) *M. conjunctiva*, the Australian *M. parki* Suárez-Morales & McKinnon, 2025, and (4) the Caribbean *M. mahahualensis* Suárez-Morales, 2022. Overall, *Monstrilla mariaeugeniae* can be easily incorporated to this species group because it shares with them key distinctive characters including: the first antennular segment unarmed, antennular segments 3 and 4 fused with constrictions marking the intersegmental divisions, and the presence of disc-like integumental windows on the antennules (Suárez-Morales 2022: figs 1C, E, F, 2B). It is thus confirmed that *M. mariaeugeniae* belongs to the *M. conjunctiva* species group and it is likely that its male will have affinities with males of the other related species of this group.

Among the known species of *Monstrilla*, the longest fifth leg endopodal lobe of the female has been described in two Baja Californian species, *M. gibbosa* and *M. hendrickxi*, and also in two Australian species, *M. janetgrieveae* and *M. latisetosa* Suárez-Morales & McKinnon, 2025. In *M. gibbosa* and *M. janetgrieveae* the endopodal lobe carries one seta and is almost as long as the outer lobe (Suárez-Morales and Palomares-García 1995: fig. 2a; Suárez-Morales and McKinnon 2025: fig. 34B). In *M. hendrickxi* the inner lobe is digitiform, slightly longer than the outer (Suárez-Morales and Velázquez-Ornelas 2024: fig. 4A), but it is unarmed as in *M. mariaeugeniae* (Fig. 22A, enp), thus different from the structure exhibited by both Australian species, *M. janetgrieveae* with one endopodal seta, and *M. latisetosa* with two endopodal setae (Suárez-Morales and McKinnon 2025: figs 37B, 39B).

## Supplementary Material

XML Treatment for Monstrilla barbata

XML Treatment for Monstrilla ciqroi

XML Treatment for Monstrilla rebis

XML Treatment for Monstrilla reidae

XML Treatment for Monstrilla elongata

XML Treatment for Monstrilla gibbosa

XML Treatment for Monstrilla mariaeugeniae

## ﻿Additional information

### Use of AI

No use of AI was reported.

### Author ORCIDs

Eduardo Suárez-Morales https://orcid.org/0000-0003-2051-8707

### Data availability

All of the data that support the findings of this study are available in the main text.
